# The p75 neurotrophin receptor attenuates secondary thalamic damage after cortical infarction by promoting angiogenesis

**DOI:** 10.1111/cns.14875

**Published:** 2024-07-28

**Authors:** Linhui Peng, Kongping Li, Dan Li, Xialin Zuo, Lixuan Zhan, Meiyan Chen, Ming Gong, Weiwen Sun, En Xu

**Affiliations:** ^1^ Department of Neurology, Institute of Neuroscience, Key Laboratory of Neurogenetics and Channelopathies of Guangdong Province and the Ministry of Education of China, The Second Affiliated Hospital Guangzhou Medical University Guangzhou Guangdong China; ^2^ Department of Neurology The Affiliated Brain Hospital, Guangzhou Medical University Guangzhou Guangdong China

**Keywords:** angiogenesis, cortical infarction, HIF‐1α, p75^NTR^, secondary damage, thalamus

## Abstract

**Background:**

Angiogenesis is crucial in neuroprotection of secondary thalamic injury after cortical infarction. The p75 neurotrophin receptor (p75^NTR^) plays a key role in activating angiogenesis. However, the effects of p75^NTR^ on angiogenesis in the thalamus after cortical infarction are largely unknown. Herein we investigate whether p75^NTR^ facilitates angiogenesis to attenuate secondary thalamic damage via activating hypoxia‐inducible factor 1α (HIF‐1α)/vascular endothelial growth factor (VEGF) pathway mediated by Von Hippel–Lindau (VHL) after distal middle cerebral artery occlusion (dMCAO).

**Methods:**

The male rat model of dMCAO was established. The effects of p75^NTR^ on the angiogenesis was evaluated using RNA‐sequencing, immunohistochemistry, western blot, quantitative real‐time polymerase chain reaction, magnetic resonance imaging, behavior tests, viral and pharmacological interventions.

**Results:**

We found that the p75^NTR^ and vessel density were decreased in ipsilateral thalamus after dMCAO. The p75^NTR^‐VHL interaction was reduced, which promoted the ubiquitination degradation of HIF‐1α and reduced VEGF expression after dMCAO. Notably, p75^NTR^ overexpression restrained the ubiquitination degradation of HIF‐1α by inhibiting VHL‐HIF‐1α interaction, further promoted angiogenesis, increased cerebral blood flow of ipsilateral thalamus and improved neurological function after dMCAO.

**Conclusion:**

For the first time, we highlighted that the enhancement of p75^NTR^‐VHL interaction promoted angiogenesis in attenuating secondary thalamic damage after dMCAO.

## Graphical abstract

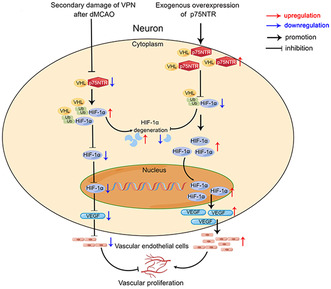

## 1 INTRODUCTION

Ischemic stroke is a leading cause of long‐term disability and mortality worldwide.^1^ Mounting evidence supports that focal cerebral infarction can cause neuronal damage in the primary infarction area, as well as nonischemic remote regions such as thalamus.^2^, ^3^, ^4^ There is a commonly accepted consensus that the prognosis of ischemic stroke is influenced by the secondary degeneration of remote regions.^5^, ^6^ We previously reported that delayed neuronal loss and glial activation occurred in the ventroposterior nucleus (VPN) of ipsilateral thalamus after distal middle cerebral artery occlusion (dMCAO) in rats, which was associated with the development of motor impairment, sensory disorder and cognitive dysfunction.^7^, ^8^, ^9^ Therefore, alleviating secondary thalamic damage is considered as an important neuroprotective strategy in the treatment of ischemic stroke.

Moreover, various clinical studies indicate that remote neuronal damage of the thalamus is related to hypoperfusion after focal cortical infarction.^2^, ^10^ Recently, promoting angiogenesis can alleviate secondary thalamic damage after ischemic stroke has been drawn attention.^11^, ^12^ However, the precise mechanisms of angiogenesis in thalamus after focal cortical infarction are largely unknown.

The p75 neurotrophin receptor (p75^NTR^), a member of tumor necrosis factor receptor superfamily, has been identified as a potential activator of angiogenesis in ischemic vascular diseases.^13^, ^14^, ^15^ Previous studies have shown that p75^NTR^ is upregulated in infarct area during ischemic stroke.^16^, ^17^ However, the alterations of p75^NTR^ in the ipsilateral VPN after dMCAO remains unclear. In hindlimb ischemia model of mice, *p75* ^*NTR*^ gene knockout impaired angiogenic function, thus leading to poor outcome of postischemic limb.^18^ Von Schack et al. found that *p75* ^*NTR*^ gene knockout resulted in the disruption of embryonic blood vessel formation, vascular rupture, extravasation of blood cells, eventually death during embryonic period.^19^ There have been reported that p75^NTR^ functions as a regulator of angiogenesis in retinal hypoxia in mice.^20^ These findings suggest that p75^NTR^ maybe a potential therapeutic target to mitigate ischemic injury by promoting angiogenesis. Whether p75^NTR^ is involved in the regulation of angiogenesis of thalamus after focal cortical infarction has not been elucidated.

It is realized that p75^NTR^ promotes angiogenesis through activation of hypoxia‐inducible factor 1α (HIF‐1α)/vascular endothelial growth factor (VEGF) pathway in retinal diseases.^21^, ^22^ HIF‐1α is a predominant regulator of cellular adaptation to hypoxia in physiology and various diseases.^23^ To regulate various biological processes such as angiogenesis, cellular proliferation and survival, HIF‐1α coordinates the response to pathological conditions by activating the transcription of a wide range of genes including VEGF. We previously confirmed that the expressions of HIF‐1α and VEGF were increased in hippocampal CA1 after transient global cerebral ischemia.^24^ The activation of neuronal HIF‐1α can attenuate ischemic damage by inducing angiogenesis after ischemic stroke.^25^ As an upstream of HIF‐1α, p75^NTR^ provides a positive feed‐forward mechanism required for HIF‐1α stabilization through undergoing hypoxia induced γ‐secretase‐dependent cleavage. Genetic loss of *p75* ^*NTR*^ dramatically reduced HIF‐1α stabilization, VEGF expression, and angiogenesis after retinal hypoxia.^20^ Whether p75^NTR^ promotes angiogenesis through regulating HIF‐1α/VEGF pathway to alleviate secondary thalamic damage after focal cortical infarction is needed to explore.

HIF‐1α has been recognized as the substrate of the E3 ubiquitin ligase Von‐Hippel Lindau (VHL) tumor suppressor gene. VHL targets the HIF‐1α protein for ubiquitination and subsequent degradation by the proteasome.^26^ Intriguingly, existing studies show that p75^NTR^ can interact directly with various E3 ubiquitin ligase family members, such as tumor necrosis factor receptor‐associated factor family (TRAF) 6 and seven in absentia homolog (Siah) 2.^20^, ^27^ Considering that VHL is a member of E3 ubiquitin ligases, we speculate that p75^NTR^ may interplay with VHL, which contributes to angiogenesis in ipsilateral VPN by regulating HIF‐1α stabilization after focal cortical infarction.

To test this hypothesis, we investigate how p75^NTR^ activates the HIF‐1α/VEGF pathway through inhibiting ubiquitination degradation of HIF‐1α induced by VHL, and then promotes angiogenesis in ipsilateral VPN of thalamus and attenuates secondary thalamic damage after dMCAO. In this project, we will provide new insight into the key role of p75^NTR^ in inducing angiogenesis in the ipsilateral thalamus to alleviate secondary thalamic damage after focal cortical infarction.

## 2 MATERIALS AND METHODS

### 2.1 Animals

All animal procedures and treatments were conducted in accordance with *Animal Research*: Reporting in vivo experiments guidelines and were approved and monitored by the Animal Care and Use Committee of Guangzhou Medical University (Guangzhou, China). All efforts had been made to minimize the suffering and the number of animals. Detailed protocols are provided in Data S1.

### 2.2 Distal middle cerebral artery occlusion model

A permanent occlusion of distal middle cerebral artery model was performed with a unipolar electrocoagulation as previously reported.^28^ Detailed protocols are provided in Data S1.

### 2.3 RNA sequencing analysis

RNA sequencing (RNA‐seq) service was offered by Beijing Genomics Institute (BGI, China). Samples from ipsilateral VPN region were collected as (*n* = 3 in each group) and immediately sent to BGI for RNA‐seq processing. Detailed protocols are provided in Data S1.

### 2.4 Immunohistochemistry

Single‐labeled immunohistochemistry was detected by the avidin–biotinperoxidase complex (ABC) method. Double‐fluorescent or triple‐fluorescent immunohistochemistry was demonstrated cell types and the exact position where p75^NTR^ or HIF‐1α were expressed as previously described.^29^ The nuclear‐associated antigen Ki‐67 (Ki67) was used to demonstrate the proliferation of vasculature.^30^ The antibodies used include rat endothelial cell antigen‐1 (RECA‐1), Laminin, Ki‐67, p75^NTR^, neuronal nuclei antigen (NeuN), glial fibrillary acid protein (GFAP), ionized calcium binding adaptor molecule‐1 (Iba‐1), HIF‐1α. Detailed protocols are provided in Data S1.

### 2.5 Western blot and co‐immunoprecipitation

Rats of each group were sacrificed at 1, 2, 3 and 4 weeks after operation respectively. Proteins of the VPN were extracted as previously described.^7^ Western blot and immunoprecipitation procedures were performed as previously described.^29^ The antibodies used include p75^NTR^, HIF‐1α, VEGF, VHL, K48‐Ub, glyceraldehyde 3‐phosphate dehydrogenase (GAPDH). Detailed protocols are provided in Data S1.

### 2.6 Adeno‐associated virus construction and administration

To improve p75^NTR^ expression, plasmids containing the sequence (ATGAGGTGGAACAGCTGCAAACAAAATAAACAAGGCGCCAACAGCCGCCCCGTGAACCAGACGCCCCCACCGGAGGGAGAGAAACTGCACAGCGACAGTGGCATCTCTGTGGACAGCCAGAGCCTGCACGACCAGCAGACCCATACGCAGACTGCCTCAGGCCAGGCCCTCAAGGGTGATGGCAACCTCTACAGTAGCCTGCCCCTGACCAAGCGTGAGGAGGTAGAGAAACTGCTCAACGGGGATACCTGGCGACATCTGGCAGGCGAGCTGGGTTACCAGCCTGAACATATAGACTCCTTTACCCACGAGGCCTGCCCAGTGCGAGCCCTGCTGGCCAGCTGGGGTGCCCAGGACAGTGCAACGCTTGATGCCCTTTTAGCCGCCCTGCGACGCATCCAGAGAGCTGACATTGTGGAGAGTCTATGCAGCGAGTCCACTGCCACGTCCCCAGTGTGA) of rat *p75* ^*NTR*^ (GenBank accession number NM_012610) and a negative control (NC) sequence (CON323) were designed by Genechem (Shanghai, China). The sequence was inserted into the hSyn promoter‐MCS‐EGFP‐3FLAG‐SV40 PolyA (GV466) adeno‐associated virus (AAV) vector. Detailed protocols are provided in Data S1.

### 2.7 Evans blue assay

The barrier function of blood vessel was measured by the area of Evans blue (EB) leakage in the brain as previously reported.^31^ Detailed protocols are provided in Data S1.

### 2.8 Magnetic resonance imaging

Magnetic resonance imaging (MRI) and the detection of cerebral blood flow (CBF) in the VPN were performed using a 9.4 T small animal MRI scanner (Bruker PharmaScan) in Jinan University, Guangzhou, Guangdong, China. Detailed protocols are provided in Data S1.

### 2.9 Pharmacologic interventions

The specific HIF‐1α inhibitor 2‐methoxyestradiol (2‐ME2) was used to determine the effects of HIF‐1α on angiogenesis and the proteasomal inhibitor MG132 was used to confirm whether p75^NTR^ regulates proteasomal degradation of HIF‐1α. The dosage and safety of 2‐ME2 and MG132 have been verified in our published studies.^24^, ^32^ Detailed protocols are provided in Data S1.

### 2.10 Quantitative real‐time polymerase chain reaction

Total RNA was extracted from the VPN of thalamus using Trizol reagent (Invitrogen, Carlsbad, CA). The mRNA levels of HIF‐1α, Pecam‐1 and Tie1 were detected. Their primer sequences and detailed protocols are provided in Data S1.

### 2.11 The evaluation of neurological function

In this study, behavioral approaches were used to evaluate neurological, sensorimotor, and cognitive functions of rats, including adhesive removal test,^33^, ^34^ beam‐walking test, Bederson scores^35^ and Morris water maze (MWM). Detailed protocols are provided in Data S1.

### 2.12 Statistical analysis

Statistical analysis was conducted with Statistical Package for Social Sciences Software for Windows, version 25.0 (SPSS, Inc., Chicago, USA). The normal distribution of data was tested by Shapiro–Wilk test. When the data were normally distributed, we used the two‐tailed t‐test for comparison between two groups. Multiple comparisons were conducted using one‐way ANOVA followed by Bonferroni's correction for multiple pairwise comparisons. When the data were normally distributed and the variances are unequal, multiple comparisons were conducted using one‐way ANOVA followed by Tamhane's T2 test for multiple pairwise comparisons. When the data were abnormally distributed and the variances were unequal, nonparametric tests were used. Mann–Whitney's *U* test was used for the comparison between two groups and Kruskal–Wallis' *H* test was conducted for multiple comparisons.

## 3 RESULTS

### 3.1 Angiogenesis in the VPN of ipsilateral thalamus after dMCAO

**FIGURE 1:**
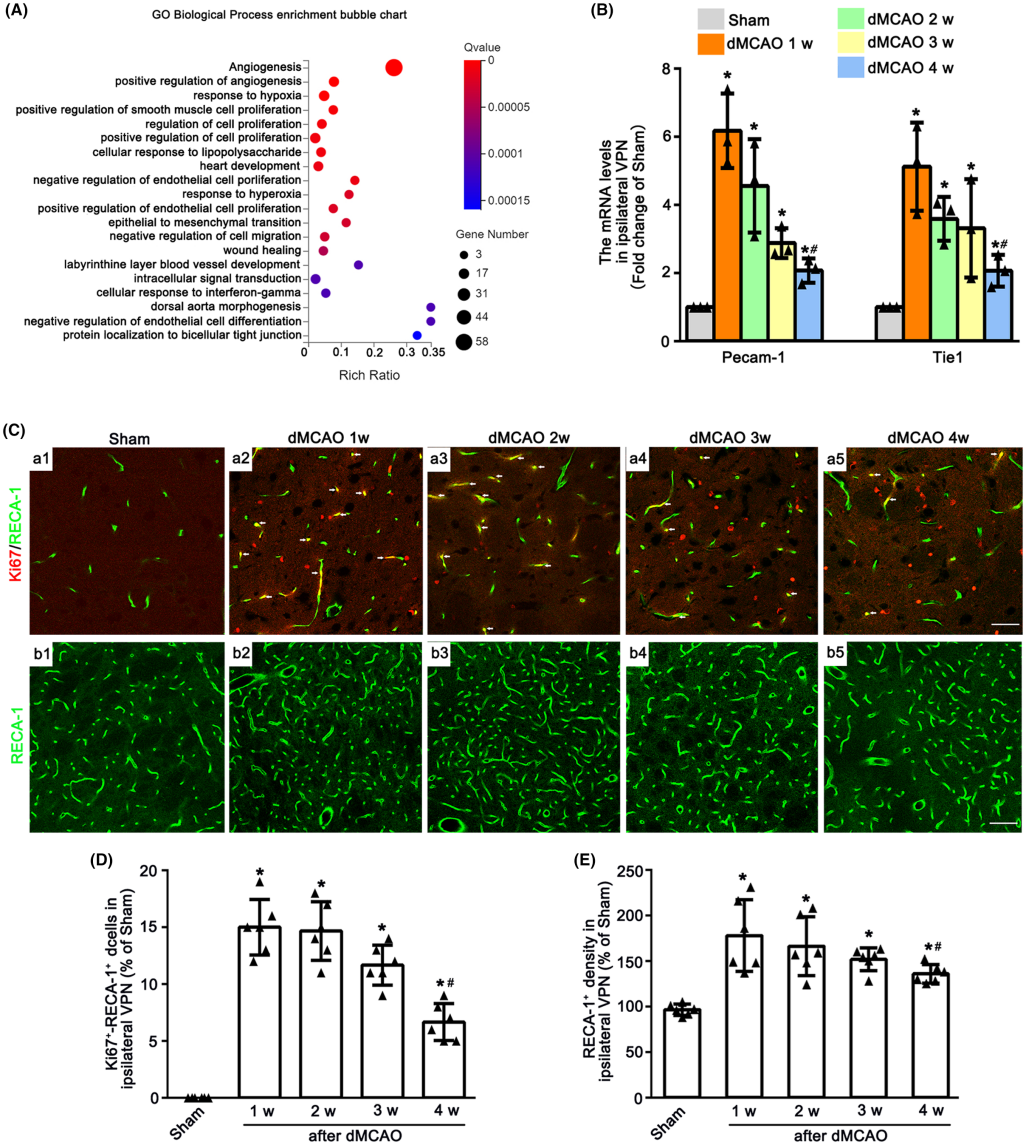
Angiogenesis in the VPN of ipsilateral thalamus after dMCAO. (A) GO biological process enrichment bubble chart in ipsilateral VPN of Sham and dMCAO rats. (B) The mRNA levels of *Pecam‐1* and *Tie1* in each group. Each bar represents the mean ± SD. *p < 0.05 versus Sham group, ^#^ *p* < 0.05 versus dMCAO 1 w group (*n* = 3 in each group). (C) Double‐staining of Ki67 (red) with RECA‐1 (green) in ipsilateral VPN between Sham and dMCAO 1–4 w. Scale bar: a1–a5: 25 μm; b1–b5: 75 μm. (D) Quantitative analysis of Ki67^+^‐RECA‐1^+^ cells. (E) Quantitative analysis of RECA‐1^+^ vessels density. Each bar represents the mean ± SD. *p < 0.05 versus Sham group, ^#^ *p* < 0.05 versus dMCAO 1 w group (*n* = 6 in each group). dMCAO, distal middle cerebral artery occlusion; Ki67, anti‐nuclear‐associated antigen Ki‐67; Pecam‐1, platelet endothelial cell adhesion molecule‐1; RECA‐1, rat endothelial cell antigen‐1; Sham, sham operation; Tie1, tyrosine kinase with immunoglobulin like and EGF like domains 1; VPN, ventroposterior nucleus; w, Week.

To detect the angiogenesis in the ipsilateral VPN after dMCAO, GO enrichment analyses were firstly conducted. The typical 20 statistically significant GO terms are shown in Figure 1A. DEGs were mainly enriched in angiogenesis, positive regulation of angiogenesis, response to hypoxia etc. after 2 weeks of dMCAO. The mRNA levels of Pecam‐1 and Tie1 in the VPN of ipsilateral thalamus at 1–4 weeks after dMCAO were upregulated when compared with Sham group (Figure 1B). To identify the angiogenesis in ipsilateral VPN after dMCAO, immunofluorescence assay was used. The number of Ki67^+^‐RECA‐1^+^ cells and the density of RECA‐1^+^ vessels were markedly increased at 1–4 weeks after dMCAO in contrast to Sham group (Figure 1C–E). It was worth noting that the mRNA levels of Pecam‐1 and Tie1, the number of Ki67^+^‐RECA‐1^+^ cells and vessel density at 4 weeks were decreased when compared with 1 week after dMCAO. These observations suggested that the angiogenesis in the ipsilateral thalamus occurred after dMCAO.

### 3.2 The downregulation of p75^NTR^ in the VPN of ipsilateral thalamus after dMCAO

**FIGURE 2:**
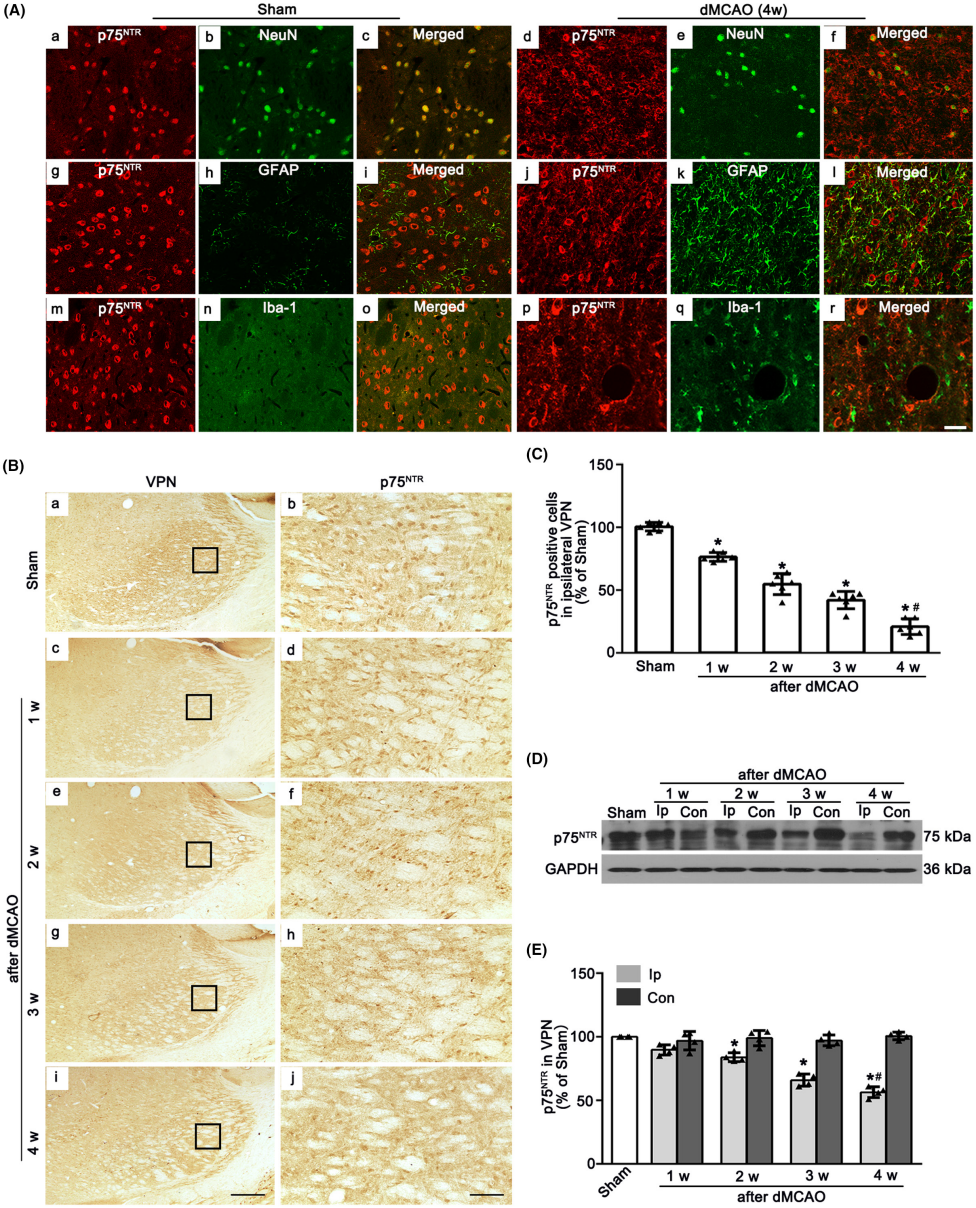
The spatial and temporal expression profiles of p75^NTR^ in the VPN of ipsilateral thalamus after dMCAO. (A) Double‐staining of p75^NTR^ (red) with NeuN^+^ neurons (green), GFAP^+^ astrocytes (green) and Iba‐1^+^ microglia (green) in ipsilateral VPN at 4 w after dMCAO. Scale bar: 25 μm. (B) Immunohistochemistry of p75^NTR^ in ipsilateral VPN of the Sham and dMCAO animals. Representative images show Sham group (a, b) and 1–4 w after dMCAO groups (c–j). Scale bar: 250 μm (a, c, e, g, i) and 50 μm (b, d, f, h, j). (C) Quantitative analyses of p75^NTR^‐positive cells in ipsilateral VPN. Each bar represents the mean ± SD. *p < 0.05 versus Sham group (*n* = 6 in each group), ^#^ *p* < 0.05 versus dMCAO 1 w group. (D) Western blot shows p75^NTR^ expression in ipsilateral VPN of the Sham and dMCAO animals. (E) Quantitative analysis of p75^NTR^ level relative to GAPDH. Each bar represents the mean ± SD. *p < 0.05 versus Sham group (*n* = 4 in each group). con, contralateral; dMCAO, distal middle cerebral artery occlusion; GAPDH, glyceraldehyde 3‐phosphate dehydrogenase; GFAP, glial fibrillary acidic protein; Iba‐1, ionized calcium‐binding adaptor molecule 1; ip, ipsilateral; NeuN, neuronal nuclei; p75^NTR^, p75 neurotrophin receptor; Sham, sham operation; VPN, ventroposterior nucleus; w, week.

To identify the cell types in which p75^NTR^ is expressed, immunofluorescence labeling of p75^NTR^ with NeuN, GFAP and Iba‐1 were measured in ipsilateral VPN after Sham or dMCAO. In Sham rats, p75^NTR^ was expressed in NeuN‐labeled cells. No colocalization of p75^NTR^ with GFAP or Iba‐1 was found, indicating that p75^NTR^ was predominantly localized in neurons. However, in dMCAO 4 weeks, p75^NTR^ was predominantly colabeled with NeuN, a few with GFAP (Figure 2A). These data revealed that p75^NTR^ was abundant in neurons in the ipsilateral VPN, whereas ischemic insult would cause the expression of p75^NTR^ in astrocytes. Most p75^NTR+^ cells from Sham and dMCAO rats had round nuclei with a granular appearance (Figure 2B). The quantitative analysis showed that as time went on, the number of p75^NTR+^ cells were significant decreased after dMCAO 1–4 weeks when compared with the Sham rats (Figure 2C). Similarly, in the western blot analysis, the expression of p75^NTR^ in the ipsilateral VPN was gradually reduced after dMCAO 2–4 weeks (Figure 2D,E).

### 3.3 Neuronal‐targeted p75^NTR^ overexpression enhances angiogenesis in the VPN of ipsilateral thalamus after dMCAO

**FIGURE 3:**
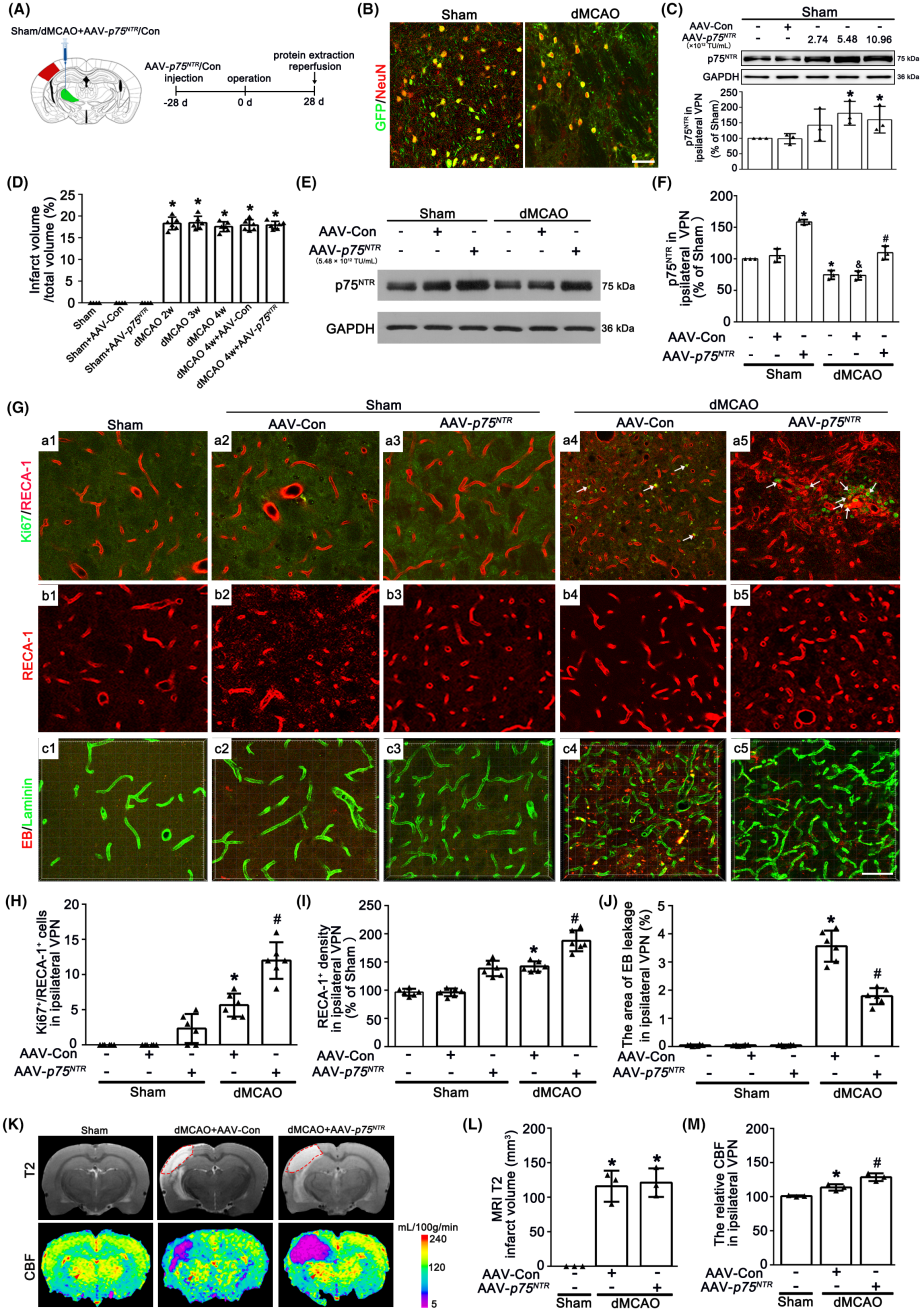
The effects of neuronal‐targeted p75^NTR^ overexpression on the angiogenesis in the VPN of ipsilateral thalamus after dMCAO. (A) Design of experiments in which rats were stereotaxically injected with *p75* ^*NTR*^ adeno‐associated virus vectors in ipsilateral VPN and subjected to either Sham or dMCAO. (B) Representative photomicrographs show the co‐localization of GFP (green) and NeuN (red) in ipsilateral VPN from Sham and dMCAO animals with AAV‐*p75* ^*NTR*^ injection. Scale bar: 75 μm. (C) Western blot shows the expression of p75^NTR^ in ipsilateral VPN of Sham rats with or without AAV‐*p75* ^*NTR*^ administration. Each bar represents the mean ± SD. *p < 0.05 versus AAV‐Con group at 4 w after dMCAO (*n* = 3 in each group). (D) Quantitative analysis of the relative infarct volumes. Each bar represents the mean ± SD. *p < 0.05 versus Sham group (*n* = 6 in each group). (E) Western blot shows p75^NTR^ expression in ipsilateral VPN in Sham or dMCAO 4 w rats injected with AAV‐Con or AAV‐*p75* ^*NTR*^. (F) Quantitative analysis of p75^NTR^ level relative to GAPDH. Each bar represents the mean ± SD. *p < 0.05 versus Sham group. ^&^ *p* < 0.05 versus Sham+AAV‐Con group. ^#^ *p* < 0.05 versus dMCAO 4 w + AAV‐Con group (*n* = 3 in each group). (G) Double‐staining of Ki67 (green) with RECA‐1 (red) in the ipsilateral VPN in Sham or dMCAO 4 w rats injected with AAV‐Con or AAV‐*p75* ^*NTR*^ vectors (arrows); c1–c5: EB (red) with Laminin (green) in the ipsilateral VPN in Sham or dMCAO 4 w rats injected with AAV‐Con or AAV‐*p75* ^*NTR*^ vectors. Scale bar: 25 μm. (H, I) Quantitative analyses of Ki67^+^‐RECA‐1^+^ cells and RECA‐1^+^ vessel density. (J) Quantitative analyses of the area of EB leakage in the ipsilateral VPN. Each bar represents the mean ± SD. *p < 0.05 versus Sham+AAV‐Con group. ^#^ *p* < 0.05 versus dMCAO 4 w + AAV‐Con group (*n* = 6 in each group). (K) Representative images of the MRI (T2 phase) and CBF in the ipsilateral VPN (red arrow) at 4 w after dMCAO. (L) Quantitative analyses of the infarct volume at dMCAO 4 w. (M) Quantitative analyses of the CBF in the ipsilateral VPN at dMCAO 4 w. Each bar represents the mean ± SD. *p < 0.05 versus Sham group. ^#^ *p* < 0.05 versus dMCAO 4 w + AAV‐Con group (*n* = 3 in each group). CBF, cerebral blood flow; dMCAO, distal middle cerebral artery occlusion; EB, evans blue; GAPDH, glyceraldehyde 3‐phosphate dehydrogenase; GFP, green fluorescent protein; Ki67, Anti‐nuclear‐associated antigen Ki‐67; MRI, magnetic resonance imaging; NeuN, neuronal nuclei; p75^NTR^, p75 neurotrophin receptor; RECA‐1, rat endothelial cell antigen‐1; Sham, sham operation; VPN, ventroposterior nucleus; w, week.

To verify the crucial role of neuronal p75^NTR^ in angiogenesis, we utilized AAV particles carrying a Syn1 promoter‐driven construct to transfer p75^NTR^ into the thalamus of rats treated with or without dMCAO. First, to ensure the efficacy of virus transfection, AAV‐*p75* ^*NTR*^ or AAV‐Con was stereotactically administered to the left VPN of thalamus 4 weeks prior to dMCAO (Figure 3A). Confocal images showed that green fluorescent protein (GFP)‐tagged AAV‐*p75* ^*NTR*^ was found to colocalize with NeuN positive cells in the ipsilateral VPN in Sham and dMCAO 4 weeks (Figure 3B), but not co‐labeled with GFAP or Iba‐1 positive cells (Figure S1A). The expression of p75^NTR^ significantly increased after AAV‐*p75* ^*NTR*^ administration at the dosage of 5.48 × 10^12^ TU/mL or 10.96 × 10^12^ TU/mL in Sham rats. However, there was no overexpressed effect of AAV‐*p75* ^*NTR*^ administration at the dosage of 2.74 × 10^12^ TU/mL (Figure 3C). Thus, the dosage of 5.48 × 10^12^ TU/mL was selected in the following experiments. No significant differences in infarct volume computed from NeuN/DAB‐staining sections were found at 2–4 weeks after dMCAO (Figures 3D and S1B). AAV‐*p75* ^*NTR*^ treatment substantially increased the expression of p75^NTR^ in the ipsilateral VPN in either Sham or dMCAO 4 weeks, when compared with that treated with AAV‐Con (Figure 3E,F). Notably, it is worth noting that the number of Ki67^+^/RECA‐1^+^ cells of AAV‐*p75* ^*NTR*^‐treated was significantly elevated compared with the AAV‐Con after dMCAO (Figure 3Ga1–a5,H). The density of RECA‐1‐labeled vessels treated with AAV‐*p75* ^*NTR*^ in the ipsilateral VPN after dMCAO was significantly higher than that with AAV‐Con (Figure 3Gb1–b5,I). Furthermore, the area of EB leakage in the ipsilateral VPN was reduced in the AAV‐*p75* ^*NTR*^‐treated dMCAO group (Figure 3Gc1–c5,J). The neuronal‐targeted p75^NTR^ overexpression could increase CBF in the ipsilateral thalamus, but not reduce cortical infarct volume at 4 weeks after dMCAO (Figure 3K–M), suggesting that the angiogenesis enhanced by neuronal‐targeted p75^NTR^ overexpression is functional.

### 3.4 Neuronal‐targeted p75^NTR^ overexpression decreases the ubiquitination of HIF‐1α mediated by VHL in the VPN of ipsilateral thalamus after dMCAO

**FIGURE 4:**
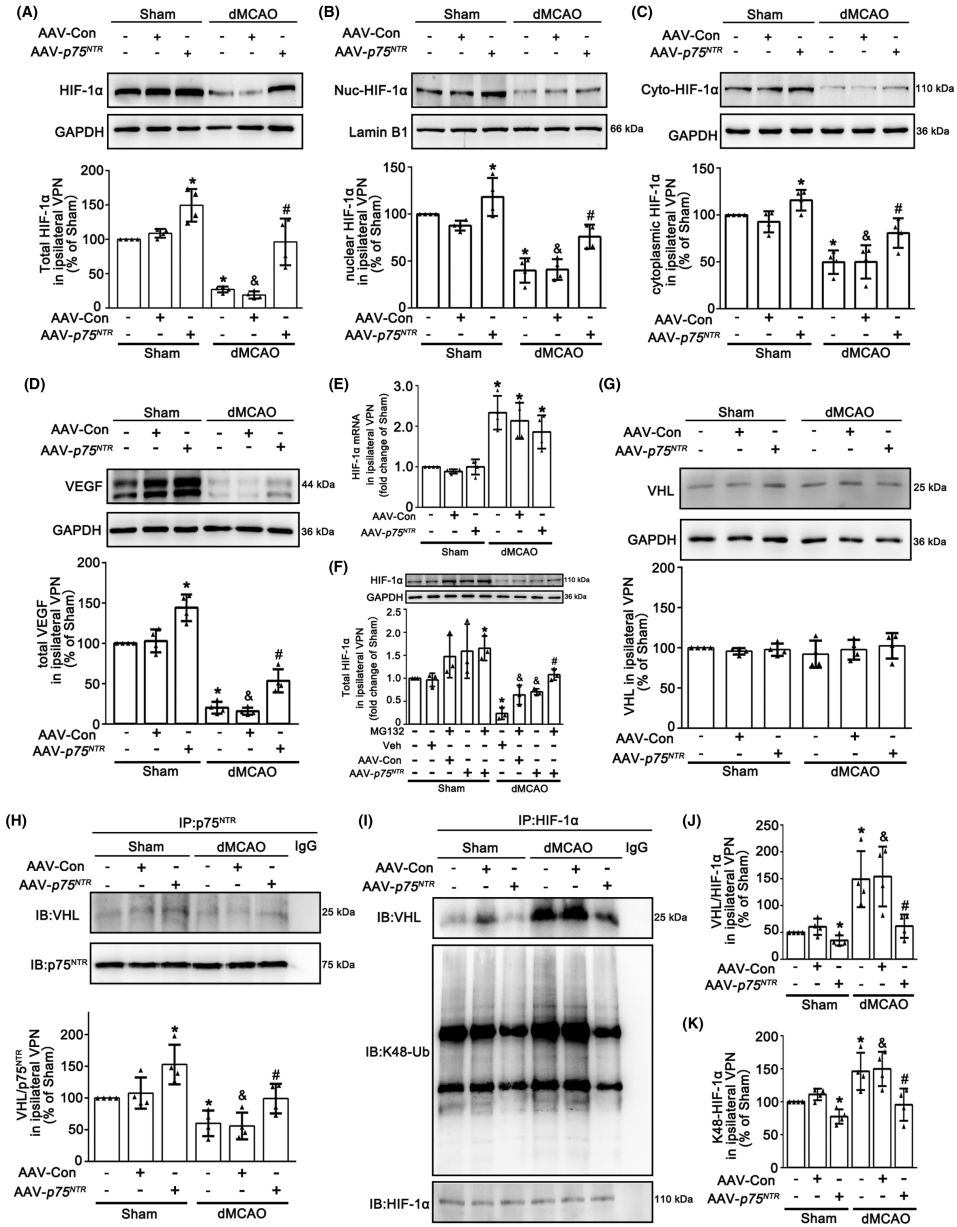
Neuronal‐targeted p75^NTR^ overexpression enhances HIF‐1α stabilization through VHL. (A) The total level of HIF‐1α in ipsilateral VPN in Sham and dMCAO 4 w rats with AAV‐Con or AAV‐*p75* ^*NTR*^ treatment. (B–D) Representative western blot, and quantitative analyses of nuclear (B) and cytoplasmic HIF‐1α (C) and VEGF (D) in ipsilateral VPN in Sham and dMCAO 4 w rats with AAV‐Con or AAV‐*p75* ^*NTR*^ treatment. Each bar represents the mean ± SD. *p < 0.05 versus Sham+AAV‐Con group, ^#^ *p* < 0.05 versus dMCAO 4 w + AAV‐Con group (*n* = 4 in each group). (E) RT‐qPCR analysis of HIF‐1α mRNA in ipsilateral VPN in the Sham and dMCAO 4 w rats with AAV‐Con or AAV‐*p75* ^*NTR*^ treatment. Each bar represents the mean ± SD. *p < 0.05 versus Sham group (*n* = 4 in each group). (F) Western blot shows HIF‐1α expression in ipsilateral VPN in Sham or dMCAO 4 w rats injected with AAV‐Con, AAV‐*p75* ^*NTR*^, Veh or MG132. Quantitative analyses of HIF‐1α levels in animals treated with or without AAV‐Con, AAV‐*p75* ^*NTR*^, Veh or MG132. Data are expressed as percentage of value of Sham animals. Each bar represents the mean ± SD. *p < 0.05 versus Sham group. ^&^ *p* < 0.05 versus dMCAO 4 w + AAV‐Con group. ^#^ *p* < 0.05 versus dMCAO 4 w + AAV‐*p75* ^*NTR*^ or dMCAO 4 w + AAV‐Con+MG132 group (*n* = 3 in each group). (G) The levels of VHL of input was detected by western blot. Quantitative analyses of VHL levels in rats treated with or without AAV‐*p75* ^*NTR*^ (*n* = 4 in each group). (H) Immunoprecipitation assay shows the level of VHL in ipsilateral VPN in Sham and dMCAO groups with AAV‐Con or AAV‐*p75* ^*NTR*^ treatment. P75^NTR^ was immunoprecipitated by anti‐p75^NTR^ antibody. IgG antibody was used as a negative control. (I) Immunoprecipitation assay shows the levels of VHL and K48‐Ub of HIF‐1α in ipsilateral VPN in Sham and dMCAO groups with AAV‐Con or AAV‐*p75* ^*NTR*^ treatment. HIF‐1α was immunoprecipitated by anti‐HIF‐1α antibody. IgG antibody was used as a negative control. The levels of VHL and K48‐Ub were detected by western blot. (J) Quantitative analysis of VHL level relative to HIF‐1α. (K) Quantitative analysis of K48‐Ub level relative to HIF‐1α. Data are expressed as percentage of value of Sham animals. Each bar represents the mean ± SD. *p < 0.05 versus Sham group, ^&^ *p* < 0.05 versus Sham+AAV‐Con group. ^#^ *p* < 0.05 versus dMCAO 4 w + AAV‐Con group (*n* = 4 in each group). dMCAO, distal middle cerebral artery occlusion; GAPDH, glyceraldehyde 3‐phosphate dehydrogenase; HIF‐1α, hypoxia‐inducible factor 1α; IP, immunoprecipitation; p75^NTR^, p75 neurotrophin receptor; Sham, sham operation; Ub, ubiquitination; VEGF, vascular endothelial growth factor; Veh, Vehicle; VHL, Von Hippel‐Lindau; VPN, ventroposterior nucleus; w, week.

Since HIF‐1α functions as a transcription factor that needs to translocate to the nucleus,^36^ we measured total, nuclear and cytoplasmic HIF‐1α protein levels respectively in the ipsilateral VPN after dMCAO. An obvious decrease of total, nuclear and cytoplasmic HIF‐1α was confirmed at 4 weeks after dMCAO. It is worth noting that neuronal‐targeted p75^NTR^ overexpression significantly increased the levels of HIF‐1α, as well as VEGF (Figure 4A–D), indicating HIF‐1α could be translocated from the cytoplasm into the nucleus, to promote VEGF transcription and expression. To further elucidate the cause of HIF‐1α reduction after dMCAO, the mRNA level of HIF‐1α in the ipsilateral VPN was measured. We found that HIF‐1α mRNA in the ipsilateral VPN was highly expressed in dMCAO with or without p75^NTR^ overexpression (Figure 4E). Then, to investigate whether the degradation of HIF‐1α in the ipsilateral VPN after dMCAO was mediated by the proteasomal degradation pathway, rats were treated with proteasome inhibitor MG132 intraperitoneally. Western blot analysis showed that when compared with vehicle treatment, HIF‐1α expression increased in Sham and dMCAO groups with MG132. In addition, there was an enhanced effect on the HIF‐1α expression when p75^NTR^ overexpression combined with MG132 (Figure 4F). To explore whether p75^NTR^ activates the HIF‐1α/VEGF pathway through inhibiting ubiquitination degradation of HIF‐1α induced by VHL, we detected the level of VHL and the p75^NTR^ ‐VHL interaction. Although there was no difference of VHL expression in the ipsilateral VPN after dMCAO with or without p75^NTR^ overexpression (Figure 4G), co‐immunoprecipitation assay showed that when compared with Sham, the p75^NTR^‐VHL interaction was obviously decreased in the ipsilateral VPN after dMCAO, which was enhanced by AAV‐*p75* ^*NTR*^ treatment (Figure 4H).

To illustrate the functional significance of alteration in p75^NTR^‐VHL interaction, we further examined the interaction between VHL and HIF‐1α. As shown in Figure 4I–K, obvious increases in the VHL‐HIF‐1α interaction and K48 ubiquitination of HIF‐1α were observed, which were reversed by AAV‐*p75* ^*NTR*^ treatment, suggesting that the decrease in HIF‐1α level in the ipsilateral VPN after dMCAO resulted from excessive ubiquitin‐proteasomal degradation.

### 3.5 Neuronal‐targeted p75^NTR^ overexpression promotes angiogenesis in the VPN of ipsilateral thalamus and offers neuroprotection via activating HIF‐1αafter dMCAO

**FIGURE 5:**
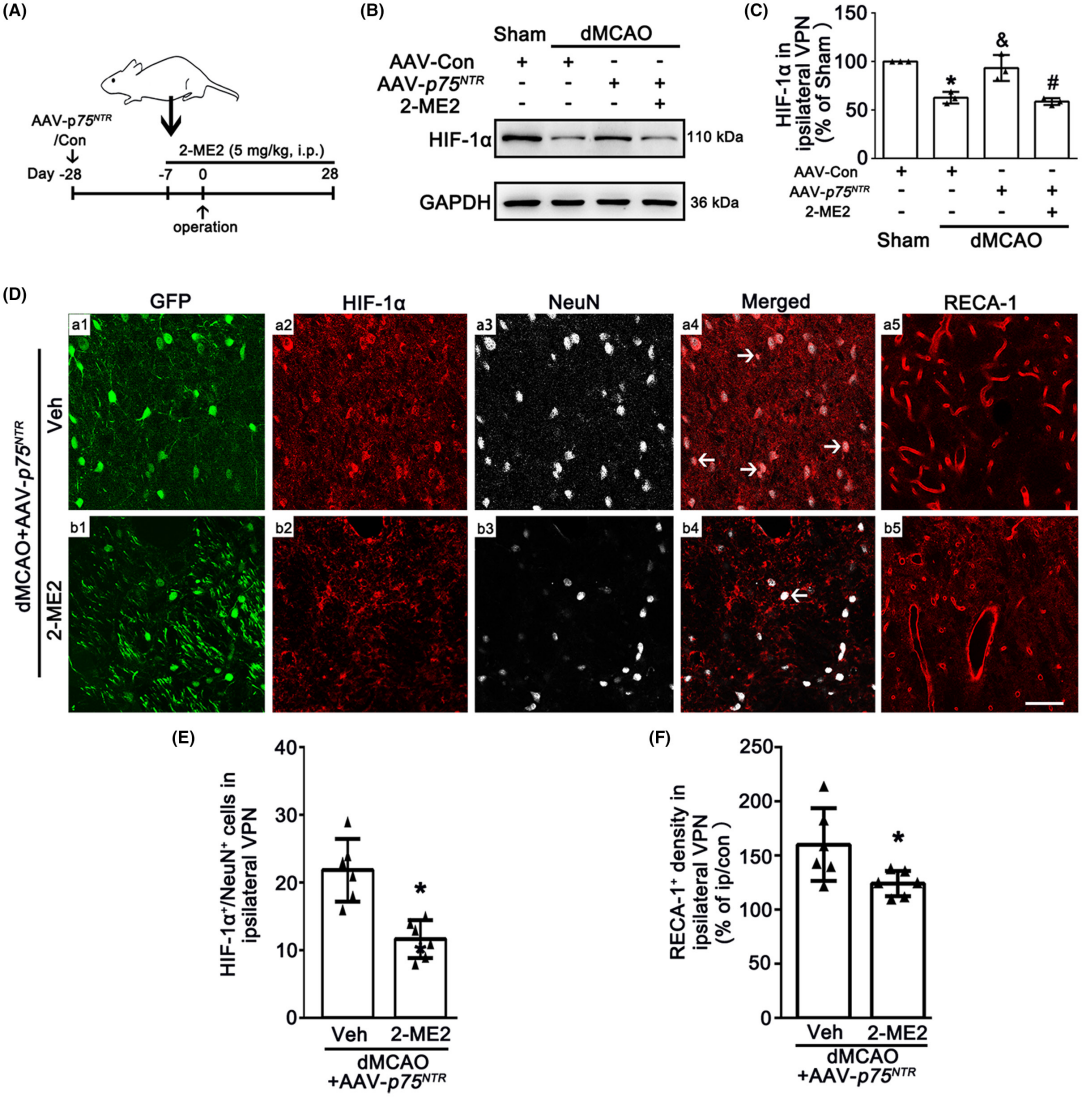
HIF‐1α inhibitor reduces the angiogenesis in the VPN of ipsilateral thalamus after dMCAO. (A) Experimental timeline in the neuronal‐targeted p75^NTR^ overexpression animals received either 2‐ME2 or Veh every day for 7 d before dMCAO until 28 days after dMCAO. (B) Western blot shows HIF‐1α expression in ipsilateral VPN after dMCAO with or without 2‐ME2 administration. (C) Quantitative analysis of HIF‐1α level relative to GAPDH. Each bar represents the mean ± SD. *p < 0.05 versus Sham group, ^&^ *p* < 0.05 versus dMCAO 4 w + AAV‐Con group, ^#^ *p* < 0.05 versus dMCAO 4 w + AAV‐*p75* ^*NTR*^ group (*n* = 4 in each group). (D) Representative photomicrographs show the triple‐staining of GFP (green), HIF‐1α (red) and NeuN (gray) and their spatial distribution along RECA‐1^+^ vessels (red) in ipsilateral VPN from dMCAO 4 w + AAV‐*p75* ^*NTR*^ animals with or without 2‐ME2 administration. (E) Quantitative analysis of HIF‐1α^+^‐NeuN^+^ cells. (F) Quantitative analysis of RECA‐1^+^ vessels density. Data are expressed as mean ± SD. *p < 0.05 versus dMCAO 4 w rats with AAV‐*p75* ^*NTR*^ + Veh (*n* = 6 in each group). 2‐ME2, 2‐methoxyestradiol; dMCAO, distal middle cerebral artery occlusion; GAPDH, glyceraldehyde 3‐phosphate dehydrogenase; GFP, green fluorescent protein; HIF‐1α, hypoxia‐inducible factor 1α; NeuN, neuronal nuclei; p75^NTR^, p75 neurotrophin receptor; RECA‐1, rat endothelial cell antigen‐1; Sham, sham operation; Veh, Vehicle; VPN, ventroposterior nucleus; w, week.

To further confirm whether neuronal‐targeted p75^NTR^ overexpression promotes angiogenesis via activating the HIF‐1α, rats were treated with 2‐ME2 intraperitoneally (Figure 5A). As expected, 2‐ME2 reversed the upregulation of HIF‐1α mediated by p75^NTR^ overexpression (Figure 5B,C). Immunofluorescence assay showed that HIF‐1α expression in neurons overexpressed p75^NTR^ and RECA‐1^+^ vessel density were decreased in 2‐ME2‐treated rats compared with vehicle group (Figure 5D–F). These observations suggest that 2‐ME2 abolishes the beneficial effects of p75^NTR^ overexpression on the angiogenesis in the ipsilateral VPN through inhibiting HIF‐1α after dMCAO.

**FIGURE 6:**
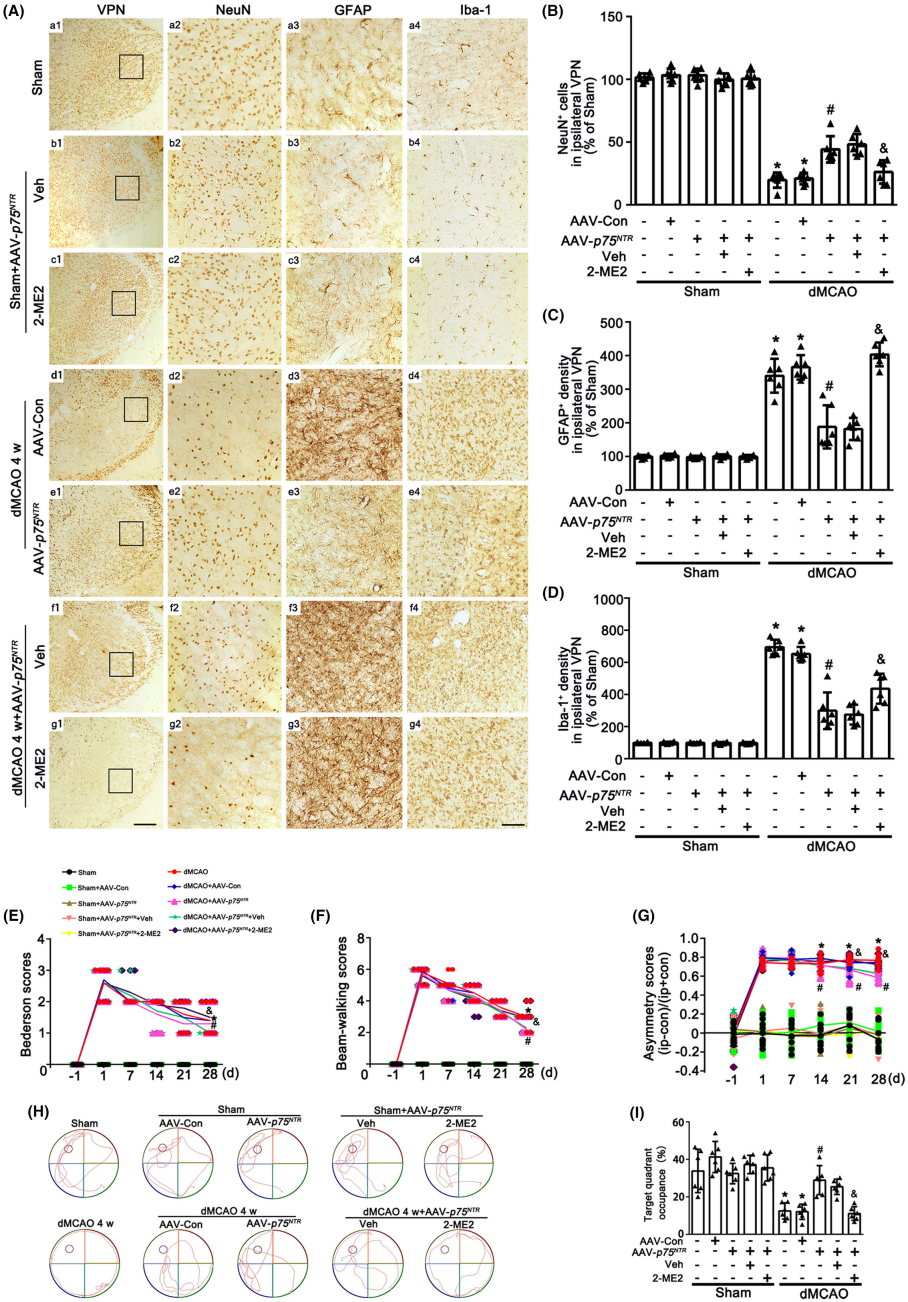
Neuronal‐targeted p75^NTR^ overexpression attenuates secondary neuronal damage of ipsilateral thalamus and improves neurological functions after dMCAO. (A) Representative microphotographs of immunostaining of NeuN, GFAP, and Iba‐1 in ipsilateral VPN. Scale bar: a1, b1, c1, d1, e1, f1, g1: 250 μm; a2–a4, b2–b4, c2–c4, d2–d4, e2–e4, f2–f4, g2–g4: 75 μm. (B–D) Quantitative analyses of NeuN positive cells (B), and GFAP (C) and Iba‐1 (D) positive density were showed from (A) in ipsilateral VPN. Data are expressed as mean ± SD. *p < 0.05 versus Sham group; ^#^ *p* < 0.05 versus dMCAO+AAV‐Con animals; ^&^ *p* < 0.05 versus dMCAO rats with AAV‐*p75* ^*NTR*^ + Veh (*n* = 6 in each group). (E–G) Neurological functions were measured on day 1 before dMCAO and on day 1, 7, 14, 21, and 28 after dMCAO with or without AAV‐Con, AAV‐*p75* ^*NTR*^, Veh or MG132 (*n* = 10 in each group). Quantitative analyses of Bederson's scores (E), beam‐walking scores (F), and asymmetry scores in adhesive removal test (G). Data are expressed as mean ± SD. *p < 0.05 dMCAO 4 w versus Sham group; ^#^ *p* < 0.05 dMCAO 4 w + AAV‐*p75* ^*NTR*^ versus dMCAO 4 w + AAV‐Con group; ^&^ *p* < 0.05 dMCAO 4 w + AAV‐*p75* ^*NTR*^ versus dMCAO 4 w + AAV‐*p75* ^*NTR*^ + Veh. (H) The representative swimming path of rats in each group. (I) The percentage of time spent in the target quadrant to total time (30 s) was recorded at 4w after dMCAO. Data are expressed as the mean ± SD. *p < 0.05 versus Sham group; ^#^ *p* < 0.05 versus dMCAO 4 w + AAV‐Con group; ^&^ *p* < 0.05 versus dMCAO 4 w rats with AAV‐*p75* ^*NTR*^ + Veh (*n* = 6 in each group). 2‐ME2, 2‐methoxyestradiol; dMCAO, distal middle cerebral artery occlusion; GFAP, glial fibrillary acidic protein; Iba‐1, ionized calcium‐binding adaptor molecule 1; NeuN, neuronal nuclei; p75^NTR^, p75 neurotrophin receptor; Sham, sham operation; Veh, Vehicle; VPN, ventroposterior nucleus; w, week.

To provide the evidence that the angiogenesis mediated by neuronal‐targeted p75^NTR^ was involved in the neuroprotection against secondary thalamic damage at 4 weeks after dMCAO, we further examined the alteration in neuronal loss, glial activation and neurological outcome. As shown in Figures 6A–D and S1C, no obvious neuronal damage or glial activation was found in Sham rats either with AAV‐Con, AAV‐*p75* ^*NTR*^ or 2‐ME2. As expected, the p75^NTR^ overexpression led to increasing number of NeuN^+^ cells and decreasing positive densities both GFAP and Iba‐1 cells in the ipsilateral VPN compared with AAV‐Con after dMCAO. However, compared with AAV‐*p75* ^*NTR*^ group, the increased NeuN^+^ cells and the decreased densities of GFAP and Iba‐1 were reversed by the 2‐ME2 treatment.

To assess the neurological functional outcome with or without p75^NTR^ overexpression after dMCAO, neurobehavioral assessment of rats was conducted. Rats in AAV‐*p75* ^*NTR*^ group exhibited lower scores in the Bederson and the beam‐walking tests than those in the AAV‐Con group after 4 weeks of dMCAO (Figure 6E,F). The mean time to remove the adhesive from the forepaws was significantly shorter in the AAV‐*p75* ^*NTR*^ group than in AAV‐Con group from 2 to 4 weeks of dMCAO (Figure 6G). Nevertheless, compared with AAV‐*p75* ^*NTR*^ group, 2‐ME2 treatment inhibited the protective effect of neurological function mediated by AAV‐*p75* ^*NTR*^ (Figure 6E–G). Then, we used MWM to assess the cognitive function of rats. After 5 days spatial orientation training, all rats improved their capacity to find the platform. As for the probe trial on day 6, rats treated with AAV‐*p75* ^*NTR*^ had longer stays in the target quadrant when compared with dMCAO with AAV‐Con. With 2‐ME2 treatment the cognitive functions of rats were observably impaired in the dMCAO group with AAV‐*p75* ^*NTR*^ (Figure 6H,I). These results suggested that neuronal‐targeted p75^NTR^ overexpression alleviates secondary thalamic damage, thereby improving neurological functions after dMCAO.

## 4 DISCUSSION

Our study confirms that p75^NTR^ plays a critical role in modulating angiogenesis in the VPN of ipsilateral thalamus after dMCAO. The expression of p75^NTR^ and the interaction of p75^NTR^‐VHL were decreased in the ipsilateral VPN after dMCAO, which in turn, increased the VHL‐HIF‐1α interaction, promoted the degradation of HIF‐1α via ubiquitin proteasome pathway, downregulated the expression of VEGF and inhibited angiogenesis, ultimately leading to secondary thalamic damage. Instead, neuronal‐targeted p75^NTR^ overexpression enhanced the interaction of p75^NTR^‐VHL and upregulated the expression of HIF‐1α and VEGF in the ipsilateral VPN through inhibiting HIF‐1α ubiquitination degradation mediated by VHL, which facilitates angiogenesis and increases CBF, consequently alleviating secondary thalamic damage after dMCAO.

Focal cortical infarction can cause delayed and selective neuronal loss in the ipsilateral thalamus that connects with the primary ischemia site.^11^, ^37^ In ischemic stroke patients and experimental model, the hypoperfusion in the ipsilateral thalamus of non‐ischemic remote brain regions is associated with delayed neuronal loss.^2^, ^11^, ^38^ Patients with ipsilateral thalamic blood flow decrease have an inferior outcome after stroke.^39^, ^40^ Improving angiogenesis may create a permissive perfusion microenvironment for the recovery of these patients. Similar with other studies,^11^, ^12^ our results showed that compared with Sham, the mRNA levels of Pecam‐1 and Tie1, the vascular density and proliferation were increased in the ipsilateral VPN at 1–4 weeks after dMCAO. However, these results were lower at 4 weeks than those at 1 week after dMCAO, implying that the compensation for angiogenesis in the ipsilateral thalamus was reduced at 4 weeks after dMCAO. Therefore, improving angiogenesis may be a promising therapeutic strategy for secondary thalamic damage after cortical infarction.

To date, the mechanism of angiogenesis inhibition in ipsilateral thalamus after dMCAO has not been fully elucidated. Previous studies focused on the central role that p75^NTR^ promotes angiogenesis in the development of peripheral vascular system^18^, ^19^ and ischemic ocular disorders.^41^ Whether p75^NTR^ modulates angiogenesis during ischemic stroke remains unclear. There have been observations demonstrated that p75^NTR^ was expressed both in neurons and astrocytes in the central nervous system,^42^, ^43^ but p75^NTR^ acts differently on them in diverse pathological conditions. For example, neuronal p75^NTR^ promotes vascularization in ischemic ocular disorders,^20^ whereas astrocytic p75^NTR^ disrupts BBB to exacerbate ischemic brain injury.^17^ In this study, p75^NTR^ was mainly expressed in neurons in both Sham and dMCAO groups, and the expression of p75^NTR^ was decreased in the ipsilateral VPN after dMCAO. Significantly, with neuronal‐targeted overexpression of p75^NTR^, the vascular density, vascular proliferation and CBF in the ipsilateral VPN were increased at 4 weeks after dMCAO, revealing that the inhibition of thalamic angiogenesis was related to the decrease of neuronal p75^NTR^. It has been recognized that promoting angiogenesis in ischemic brain region is associated with neurological rehabilitation and longer survival.^44^, ^45^ Previous investigation has confirmed that p75^NTR^ from endothelial progenitor cell exerts a protective effect on angiogenesis in a mice model with hindlimb ischemia.^18^ Zanin et al. found that the absence of p75^NTR^ prevented the ability of brain‐derived neurotrophic factor to rescue hippocampal neurons in a trophic deprivation model, indicating that p75^NTR^ is critical for promoting neuronal survival.^46^ Our research conferred the evidence that neuronal‐targeted p75^NTR^ overexpression reduced the permeability of BBB, increased CBF and attenuated neuronal loss in the ipsilateral thalamus and improved neurological function by promoting angiogenesis rather than reducing cortical infarct volume after dMCAO.

As mentioned above, p75^NTR^ is mainly expressed in neurons. Le moan et al. demonstrated that p75^NTR^ played an important biological role in retinal angiogenesis through the regulation of HIF‐1α stabilization and VEGF expression.^20^ It is evident that HIF‐1α is mainly expressed in neurons and exerts neuroprotection against cerebral ischemia.^24^, ^47^ The activation of HIF‐1α/VEGF pathway plays an important part in the regulation of angiogenesis and neuronal survival, whereas the absence of neuronal HIF‐1α inhibited angiogenesis, aggravated neuronal death and impaired neurobehavioral function after focal cerebral ischemia in mice.^25^, ^48^, ^49^ Furthermore, neuronal VEGF can enhance angiogenesis, neurogenesis and neuroprotection in subchronic phase of MCAO‐induced stroke.^50^ Herein, we noticed a significant reduction of total, cytoplasmic and nuclear HIF‐1α as well as VEGF in the ipsilateral VPN at 4 weeks after dMCAO. Neuronal‐targeted p75^NTR^ overexpression restored cytoplasmic and nuclear HIF‐1α, and VEGF expression. Therefore, we believed that the overexpression of neuronal p75^NTR^ increased angiogenesis possibly via activating HIF‐1α/VEGF pathway, thereby facilitating neuronal survival in ipsilateral VPN after dMCAO.

Notably, HIF‐1α mRNA level was increased in ipsilateral VPN after dMCAO, which implied that the reduction in HIF‐1α was not attributable to modified transcriptional levels. Neuronal‐targeted p75^NTR^ overexpression did not interfere with the transcription of HIF‐1α. This phenomenon let us to further investigate the potential role of p75^NTR^ in the stabilization of HIF‐1α. As the stabilization of HIF‐1α is primarily governed by the ubiquitin‐proteasome pathway,^47^, ^51^ we first explored whether the degradation of HIF‐1α in ipsilateral VPN after dMCAO was mediated by proteasomal pathway. As expected, the treatment with MG132 could reduce the degradation of HIF‐1α in ipsilateral VPN after dMCAO, indicating the involvement of proteasomal pathway in the decrease of HIF‐1α.

Since the VHL protein, an E3 ubiquitin ligase, plays an essential role in targeting HIF‐1α for ubiquitin‐proteasomal degradation and thereby inhibiting angiogenesis,^52^ we detected the level of VHL in ipsilateral VPN after dMCAO with or without AAV‐*p75* ^*NTR*^. Intriguingly, neuronal‐targeted p75^NTR^ overexpression had no impact on VHL expression in ipsilateral VPN following dMCAO. This suggests that p75^NTR^ upregulates HIF‐1α expression through another mechanism that is independent of VHL expression. There has been reported that p75^NTR^ directly interacts with E3 ubiquitin ligase Siah 2 or TRAF 6 to restrain the ubiquitination degradation of substrate proteins.^20^, ^27^ Hence, it is possible that p75^NTR^ might exert regulatory control over the ubiquitin‐proteasomal degradation of HIF‐1α through its interaction with VHL. Our investigation substantiated that the attenuation of p75^NTR^‐VHL interaction subsequent to dMCAO resulted in an enhanced VHL‐HIF‐1α interaction, which facilitated the ubiquitination degradation of HIF‐1α, whereas neuronal‐targeted p75^NTR^ overexpression effectively impeded these effects. Collectively, our results implied that p75^NTR^ regulated the stability of HIF‐1α via disturbing VHL function in the ipsilateral VPN after dMCAO.

Further, to determine the participation of HIF‐1α in the angiogenesis and neuroprotection promoted by p75^NTR^ overexpression, HIF‐1α specific inhibitor 2‐ME2 was applied. The results showed that 2‐ME2 reversed upregulation of HIF‐1α mediated by p75^NTR^ overexpression, impaired the angiogenesis and aggravated secondary thalamic damage after dMCAO, indicating that the neuroprotection offered by p75^NTR^ overexpression was dependent on HIF‐1α activation. The activation of HIF‐1α by p75^NTR^ overexpression enhanced the microvasculature in ipsilateral thalamus, which would provide a better perfusion environment for maintaining neuronal metabolism, thereby improving neurological function of rats after cortical infarction.

Admittedly, a main limitation in this study was only male rats were used. Although in preclinical study male rodents have been the default model organism for many years, there has been growing recognition of the significance of sexual dimorphism in various diseases or healthy conditions. The potential sex differences in response to stroke therapies,^53^ pathological brain lipid metabolism in diet of hyperhomocysteinemia,^54^ vasomotor reactivity to carbon dioxide,^55^ the function of neurovascular coupling,^56^ mitochondrial metabolism,^57^ plastic remodeling,^58^ etc. have likely contributed to higher rates of misdiagnosis and adverse side effects from the treatments in women. Considering sexual dimorphism in response to ischemic stroke, in future both sexes should be included to improve the rigor and reproducibility of research.

In summary, the present study provides a novel insight into the mechanism by which p75^NTR^ promotes angiogenesis in the ipsilateral thalamus after dMCAO. The overexpression of neuronal p75^NTR^ hinders the binding between VHL and HIF‐1α, and prevents the degradation of HIF‐1α from ubiquitination and elevates VEGF expression, thus increasing CBF and alleviating secondary thalamic damage after dMCAO. This strategy may address both vascular repair and neuronal degeneration in the ipsilateral thalamus after cortical infarction by promoting angiogenesis.

## AUTHOR CONTRIBUTIONS

E.X., L.P. and K.L. conceived the study, designed the experiments and assembled all the figures. L.P. and K.L. performed the experiments with the assistance of D.L., X.Z., M.C., M.G. and W. S. This article was written by E.X., L.P. and K.L. L. Z. assisted in the design of this project. All authors read and approved the final version of this article.

## FUNDING INFORMATION

This work was supported by National Natural Science Foundation of China (Grant No. 81971233) and Science and Technology Program of Guangzhou, China (2024A03J0199, 2023A04J1203).

## CONFLICT OF INTEREST STATEMENT

The authors declared no potential conflicts of interest.

## Supporting information


Data S1.

Data S2.

Figure S1.

## Data Availability

The data that support the findings of this study are available from the corresponding author upon reasonable request.
