# Supplementary material for: The p75 neurotrophin receptor attenuates secondary thalamic damage after cortical infarction by promoting angiogenesis
Source: CNS Neurosci Ther. 2024 Jul 28;30(7):e14875. doi: 10.1111/cns.14875 (PMC11284236; doi:10.1111/cns.14875)
Supplement: Supplementary file 1 — Data S1. [file CNS-30-e14875-s001.docx]

**Materials and Methods**

**Animals**

Adult male SD rats weighing 280-320 g (10-12 weeks) used in this study were supplied by Southern Medical University (Guangzhou, China). Rats were housed in a controlled environment under standard temperature (22±1℃) and a 12 hours light/dark cycle with free access to food and water in this study. Weight gain and health condition of rats are comparable between different groups.

In this study, 397 male rats were used, 1 in the Sham-operated (Sham) and 2 in the dMCAO groups died during the dMCAO procedure. In addition, 4 rats in group of dMCAO 4 weeks, 3 in group of dMCAO 4 weeks with AAV-Con and 2 in group of dMCAO 4 weeks with AAV-*p75^NTR^* without cortical infarcts after surgery were excluded.

**Distal middle cerebral artery occlusion model**

Rats were anesthetized with isoflurane (3-4%) in 100% oxygen and maintained with isoflurane (1.5-2.5%) in 100% oxygen, delivered by a nose mask (SurgiVet, Waukesha, WI, USA) during the surgical procedure. The distal striatal branch of left MCA was exposed and then occluded with a unipolar electrocoagulation device. Sham-operated (Sham) rats received the same surgical procedures except for left dMCA electrocoagulation. After surgery, neurological functional evaluations of rats were conducted. All animals performed normal feeding and drinking behaviors postoperatively.

**RNA sequencing analysis**

RNA sequencing (RNA-seq) service was offered by Beijing Genomics Institute (BGI, China). Samples from ipsilateral VPN region were collected as (n=3 in each group) and immediately sent to BGI for RNA-seq processing. The cDNA library establishment was in compliance with the BGI standard procedures. Clean reads were mapped to the Rattus_norvegicus (rats: NCBI_GCF_000001895.5). In terms of functional enrichment analysis, all differentially expressed genes (DEGs) were mapped to terms in the Gene Ontology (GO) biological process analysis. The data mining and process of making the figure were finished by the BGI in-house customized data mining system called Dr. Tom (<http://report.bgi.com>).

**Immunohistochemistry**

The rats from each group were perfused intracardially with 0.9% normal saline and 4% cold paraformaldehyde in phosphate-buffered saline (PBS, 0.01 M, pH 7.4). All brain tissues were removed quickly and postfixed in 10%, 20%, and 30% sucrose in the same fixative overnight at 4°C for cytoprotection. After post fixation, the brains were frozen under -20 °C and sliced into coronal 30-μm-thick sections with cryotome (Leica, Wetzlar, Hessen, Germany). The coronal sections that included the ventroposterior nucleus (VPN) of thalamus were selected for use into following experiments. Single-labeled immunohistochemistry was detected by the avidin-biotinperoxidase complex (ABC) method. Briefly, the sections were first treated with 3% hydrogen peroxide for 30 minutes, followed by 5% normal serum for 1 hour at ordinary temperature, and then incubated with mouse monoclonal antibody against neuronal nuclei antigen (NeuN, 1:5000; MilliporeSigma, Cat# MAB377, RRID: AB_2298772), mouse monoclonal antibody against glial fibrillary acid protein (GFAP, 1:5000; MilliporeSigma, Cat# MAB360, RRID: AB_11212597), mouse monoclonal antibody against ionized calcium binding adaptor molecule-1 (Iba-1, 1:5000; MilliporeSigma, Cat# MABN92, RRID: AB_10917271) or rabbit anti-p75^NTR^ monoclonal antibody (1:1000; Abcam,, Cat# ab52987, RRID: AB_881682) overnight at 4 °C. Then, the slides were washed with PBS for six times. Afterwards, the slides were incubated with biotinylated secondary immunoglobulin G (IgG) antibody for 2 hours at ambient temperature. After being washed with PBS for three times, the sections were subsequently soaked with ABC for 30 min at ordinary temperature. Immunopositive cells in which the reaction product was present within a clear and regular-shaped cytoplasmic or nuclear border were quantified under a light microscope with ×660 magnification. The total number of immunopositive cells in the thalamus was counted within three nonrepeated random fields (0.037 mm^2^/field×3=0.111 mm^2^ in total). The cortex infarct volume was measured by calculating the percentage of infarct volume relative to the respective contralateral hemisphere volume.

Double-fluorescent or triple-fluorescent immunohistochemistry was performed and demonstrated cell types and the exact position where p75^NTR^ or HIF-1α were expressed. NeuN, GFAP and Iba-1 were used to identify nuclei of neurons, astrocytes, and microglia, respectively. The primary antibodies used in this part included rabbit anti-rat endothelial cell antigen-1 (RECA-1; 1:200, Abcam, Cat# ab9774, RRID: AB_296613), rabbit anti-p75^NTR^ monoclonal antibody (1:200; Abcam, Cat# ab52987, RRID: AB_881682), rabbit anti-Ki-67 monoclonal antibody (1:200; Abcam, Cat# ab15580, RRID: AB_443209), rabbit anti-Laminin polyclonal antibody (1:200; MilliporeSigma, Cat# L9393, RRID: AB_477163), rabbit anti-HIF-1α monoclonal antibody (1:100; Abcam, Cat# ab179483, RRID: AB_2732807), mouse anti-NeuN (1:1000) monoclonal antibody, mouse anti-GFAP (1:1000) monoclonal antibody (1:5000), mouse anti-Iba-1 (1:1000) monoclonal antibody (1:1000). The secondary antibody included Cy3-conjugated goat anti-mouse IgG antibody (1:200; MilliporeSigma, Cat# AP124C, RRID: AB_11213281), Cy3-conjugated goat anti-rabbit IgG antibody (1:200; MilliporeSigma, Cat# AP132C, RRID: AB_92489), 488-conjugated goat anti-mouse IgG antibody (1:200; Abcam, Cat# ab150113, RRID: AB_2576208), and 488-conjugated goat anti-rabbit IgG antibody (1:200; Abcam, Cat# ab150077, RRID: AB_2630356), 647-conjugated goat anti-rabbit IgG antibody (1:200; Invitrogen, Cat# A21244, RRID: AB_2535812). After being incubated with IgG antibody, sections were washed with PBS and mounted with mounting medium containing 4′,6-diamidino-2-phenylindole (DAPI, Solarbio, Cat# S2110). Slides were analyzed with a confocal laser microscope (SP8, Leica Microsystems, Wetzlar, Hessen, Germany).

**Western blot**

Rats of each group were sacrificed at 1, 2, 3, and 4 weeks after operation. The brain tissue was cut into 2 mm-thick coronal slices using a brain matrix (Shuolinyuan Technology Co., Ltd., Beijing, China) and the VPN regions of bilateral thalamus were quickly dissected under a stereomicroscope. Proteins of the VPN were extracted. The bicinchoninic acid (BCA) method was recommended by the manufacturer (Beyotime) to detect protein concentration. The proteins of each sample were separated by sodium dodecyl sulfate-polyacrylamide gel electrophoresis (SDS-PAGE) and then transferred to polyvinylidene fluoride (PVDF) membranes (MilliporeSigma, Burlington, MA, USA). The primary antibodies used in these studies including p75^NTR^ (1:1000; Abcam, Cat# ab52987, RRID: AB_881682), HIF-1α (1:2000; Abcam, Cat# ab179483, RRID: AB_2732807), rabbit anti-VEGF (1:5000; Novus Biologicals, Cat# NB100-664, RRID: AB_10001947), rabbit anti-VHL polyclonal antibody (1:2000; Affinity Biosciences, Cat# AF6292, RRID: AB_2835142), and glyceraldehyde 3-phosphate dehydrogenase (GAPDH, 1:10000; Proteintech Group, Cat# 60004-I-Ig, RRID: AB_2107436). Compartmental expression of HIF-1α was measured on nuclear versus cytosolic proteins. The nuclear and cytosolic proteins were separated by using an NE-PER Nuclear Cytoplasmic Extraction Reagent kit (Pierce, Rockford, IL). The purity of each fraction was confirmed by measuring the expression of reporters, including Lamin B1 (1:2000; Proteintech Group, Cat# 12987-1-AP, RRID: AB_2136290) and GAPDH (1:10000; Proteintech Group, Cat# 60004-I-Ig, RRID: AB_2107436). Also, the same amount of protein (60 μg) was applied. Densitometric analysis for the quantification of the bands was performed with Image J (NIH, Bethesda, MD, United States). Relative optical densities of protein bands were calibrated with GAPDH or Lamin B1 and normalized to those in Sham-operated rats.

**Co-immunoprecipitation**

Thalamic proteins (120 μg per group) were incubated with rabbit anti-p75^NTR^ monoclonal antibody (1:50) or rabbit anti-HIF-1α monoclonal antibody (1:50) at 4°C overnight. The next day, the protein/antibody complex was added to packed protein G agarose beads (MilliporeSigma, Cat# IP05). Following 4 hours of incubation at 4℃, the complex was washed five times with PBS containing 1% Tween. Immunocomplexes were collected by centrifugation and eluted by boiling in loading buffer. The eluted protein samples were subjected to Western blotting with various antibodies including p75^NTR^ (1:5000, Abcam, Cat# ab52987, RRID: AB_881682), VHL (1:2000, Affinity Biosciences, Cat# AF6292, RRID: AB_2835142), HIF-1α (1:2000, Abcam, Cat# ab179483, RRID: AB_2732807), and rabbit anti-ubiquitin (linkage-specific K48) monoclonal antibody (1:4000; Abcam, Cat# ab140601, RRID: AB_2783797). Densitometric analysis was performed to quantify the relative precipitated protein bands, which were calibrated with the bands of p75^NTR^ or HIF-1α (ratio of bound to p75^NTR^ or HIF-1α) and normalized to those in Sham rats.

**Adeno-associated virus construction and administration**

To improve p75^NTR^ expression, plasmids containing the sequence (ATGAGGTGGAACAGCTGCAAACAAAATAAACAAGGCGCCAACAGCCGCCCCGTGAACCAGACGCCCCCACCGGAGGGAGAGAAACTGCACAGCGACAGTGGCATCTCTGTGGACAGCCAGAGCCTGCACGACCAGCAGACCCATACGCAGACTGCCTCAGGCCAGGCCCTCAAGGGTGATGGCAACCTCTACAGTAGCCTGCCCCTGACCAAGCGTGAGGAGGTAGAGAAACTGCTCAACGGGGATACCTGGCGACATCTGGCAGGCGAGCTGGGTTACCAGCCTGAACATATAGACTCCTTTACCCACGAGGCCTGCCCAGTGCGAGCCCTGCTGGCCAGCTGGGGTGCCCAGGACAGTGCAACGCTTGATGCCCTTTTAGCCGCCCTGCGACGCATCCAGAGAGCTGACATTGTGGAGAGTCTATGCAGCGAGTCCACTGCCACGTCCCCAGTGTGA) of rat *p75^NTR^* (GenBank accession number NM_012610) and a negative control (NC) sequence (CON323) were designed by Genechem (Shanghai, China). The sequence was inserted into the hSyn promoter-MCS-EGFP-3FLAG-SV40 PolyA (GV466) AAV vector. To select the highest overexpression effects of p75^NTR^, we used three doses of AAV-*p75^NTR^* at 2.74×10^12^, 5.48×10^12^ or 1.096×10^13^ v.g/mL, respectively. A total of 2 μL volume (0.2 μL virus diluted by 1.8 μL PBS) containing of particles was injected into the VPN of ipsilateral thalamus (3.6 mm posterior to bregma, 3.0 mm lateral to bregma, 6.0 mm below the dura) of rats using a 10-μL Hamilton syringe with 34-gauge needle at a flow rate of 0.2 μL/min. The rats were allowed to recover for up to 28 days to enable adequate gene expression.

**Quantitative real-time polymerase chain reaction**

Total RNA was extracted from the VPN of thalamus using Trizol reagent (Invitrogen, Carlsbad, CA). Quantitative real-time polymerase chain reaction (qRT-PCR) was performed under the standard protocol. For each sample, 1 μg of RNA was reverse transcribed into cDNA in a final volume of 20 μL with 1μL primer mix, 1μL enzyme mix and 4 μL buffer (Takara, Shiga, Japan). The primers used are as follow: HIF-1α: 5’-CGGAAACTGAAGACCAACAAC-3’ (forward), 5’-CAGAGGCAGGTAATGGAGACA-3’ (reverse);

Pecam-1: 5’-CATGGTGGAGCACAGTGGCA-3’ (forward), 5’-TGGGATGGAGCAGGACAGGTT-3’ (reverse);

Tie1: 5’-TGCGAGCCCAGTCCAAGAGA-3’ (forward), 5’-ACAGGGTAACTCAAAGGCTC-3’ (reverse);

GAPDH: 5’-ACCACAGTCCATGCCATCAC-3’ (forward), 5’-TCCATGGCGAACTGGTGGCG-3’ (reverse); (Sangon Biotech, Shanghai, China). Then, PCR was performed in an ultimate volume of 25μL with 2μL of RT product for cDNA amplification. Annealing temperature was 60 ℃. Quantitative PCRs were conducted by LightCycler Fast-Start DNA Master SYBR Green 1 kit and on a LightCycler 1.5 PCR machine (Roche Light Cycler 480, Germany). Each sample was run in triplicates. All thalamic samples were quantified against the identical standard curve, and the HIF-1α expression level was normalized to GAPDH expression level. Data were analyzed by using the comparative Ct method (2^-ΔΔ^*^Ct^*). Results were expressed as fold changes compared to Sham group.

**Evans blue assay**

Evans blue (EB) dye (2%, 4 mL/kg; Sigma-Aldrich) was intravenously administered via the tail vein at 4 weeks after dMCAO. After 1 hour of circulation, the mice were anesthetized and perfused with 0.9% normal saline, followed by 4% cold PFA in PBS (0.01 M, pH 7.4) under anesthesia with 4% isoflurane. The brains were removed quickly and post-fixed for 12 hours in 4% PFA and then cryoprotected with 10, 20, 30% sucrose in the same fixative overnight. Coronal tissue blocks were cut on a freezing microtome (Leica, Wetzlar, Hessen, Germany) into 30 µm-thick sections. The area of EB leakage in ipsilateral VPN was analysed by a confocal laser microscope (SP8, Leica Microsystems, Wetzlar, Hessen, Germany).

**Magnetic resonance imaging**

Magnetic resonance imaging (MRI) and the detection of cerebral blood flow (CBF) in the VPN were performed using a 9.4 T small animal MRI scanner (Bruker PharmaScan) in Jinan University, Guangzhou, Guangdong, China. The rats were anesthetized with 2% isoflurane administered via a nose cone, while monitoring their body temperature and respiratory rate. T2-weighted imaging (T2WI) was conducted at 4 weeks after dMCAO using the following scanning parameters: 2D fast-spin echo sequence (3500/33 ms of repetition time/echo time, 2 average), field of view (FOV)=35×35 mm^2^, slice thickness=0.8 mm, number of slices=31, matrix=256×256. Under the same scale and brain slices of dMCAO rat images, T2WI imaging was scanned and quantified using 3D slicer software (<https://www.slicer.org/>). Finally, the infarct volume was determined by 3D slicer software.

CBF was quantified using the arterial spin labeling (ASL) technique. Flow-sensitive alternating inversion recovery rapid acquisition with relaxation enhancement (FAIR-RARE) was employed for ASL, with the following parameters: 2D fast-spin echo sequence (4000/16 ms of repetition time/echo time, 1 average), FOV=25×25 mm^2^, slice thickness=1 mm, number of slices=8, and matrix=128×128. The perfusion map in the thalamus was quantified through manual delineation of the regions of interest.

**Pharmacologic interventions**

To determine the effects of HIF-1α on angiogenesis in the thalamus, the rats were treated with the specific HIF-1α inhibitor 2-methoxyestradiol (2-ME2) (5 mg/kg, intraperitoneally; Sigma Aldrich, Cat# HY-12033) or vehicle (isometric 10% dimethyl sulfoxide, DMSO) at 1 week before dMCAO until 4 weeks after dMCAO. To confirm whether p75^NTR^ regulates proteasomal degradation of HIF-1α, the rats were treated with proteasomal inhibitor MG132 (5 mg/kg, intraperitoneally; Sigma Aldrich, Cat# 3211020) at 1 week before dMCAO until 4 weeks after dMCAO. Both 2-ME2 and MG132 were dissolved in 10% DMSO.

**The evaluation of neurological function**

Behavioral tests were conducted at 1, 7, 14, 21 and 28 days after dMCAO by an investigator who was blinded to the experimental groups. All rats were acclimated to the testing environment before surgery. The adhesive removal test was performed by placing a 200 mm^2^ piece of adhesive paper sequentially onto the ipsilateral and contralateral forepaw. The performance of rats was assessed by measuring the time required to sense and remove the adhesives. The time to remove the adhesives from the forelimbs was recorded in three replicates, with each trial separated by at least 3 minutes. The Bederson scores were used to evaluate neurologic function, with forelimbs of normal rats extended towards the floor and flexion of the forelimb contralateral to the lesioned hemisphere occurring in ischemic rats. The behavioral scores were: 1, forelimbs curved with no other abnormality; 2, reduced resistance to lateral push towards the paralytic side; 3, circling to the paralyzed side when the flat ground is free to move; 4, spinning along the longitudinal axis of the body when the tail is raised. Additionally, the beam-walking test was used to assess motor balance function using a 122 cm long, 2.5 cm wide beam. Scoring was based on the following criteria: 0, falling from the beam directly; 1, unable to walk on the beam but remaining seated across it; 2, falling from the beam while walking; 3, walking along the beam, but the injured hindlimb not functioning in forward movement; 4, crossing the beam with more than 50% footslips; 5, crossing the beam with occasional footslips; 6, crossing the beam without footslips. All rats underwent three training trials per day for three days before dMCAO.

The Morris water maze (MWM) was conducted to assess cognitive function in rats. The aquatic maze consists of a circular black pool (210 cm in diameter, 60 cm in height; Taimeng Technology Co., Ltd, Chengdu, China) filled with opaque water (20 cm in depth, 23±1℃). The pool was divided into four equal-sized quadrants (Q1, Q2, Q3, and Q4), in which a transparent platform (escape platform, 10 cm in diameter) was placed 2.0 cm below the water surface in Q4.

Rats were put into the pool facing toward the wall and every starting point was used in a different order each day. Starting positions were designated as Q1, Q2, Q3 or Q4. All rats were trained to find the hidden platform within 120 seconds and then stayed on the platform for 15 seconds. If the rats failed to find the platform, it was guided to get there, and the escape latency was recorded as 120 seconds. During the training period, the platform remained in the same location. All rats were subjected to 4 consecutive trials each day at 10 minutes intervals for 5 consecutive days from the 23rd day after dMCAO. The escape latency, path length and swimming speed for each trial were recorded.

Twenty-four hours after the end of a training period, a 30 seconds probe trial without the platform was conducted to assess the long-term memory. All rats were placed in water maze at a fastened starting point (the most distance to the platform position used during the training period). The time in the target quadrant occupancy was also recorded at the 28 days after dMCAO.

All tests were almost conducted at the same time period each day to minimize variability in rat performance. The parameters for each trial were recorded by a video tracking system (SMART, Polyvalent video-tracking system, 35B73-C6C, PANLAB, Spain).
