# Supplementary material for: The p75 neurotrophin receptor attenuates secondary thalamic damage after cortical infarction by promoting angiogenesis
Source: CNS Neurosci Ther. 2024 Jul 28;30(7):e14875. doi: 10.1111/cns.14875 (PMC11284236; doi:10.1111/cns.14875)
Supplement: Supplementary file 2 — Data S2. [file CNS-30-e14875-s003.pdf]

Full unedited blot for Figure 2D p75^{NTR} and GAPDH

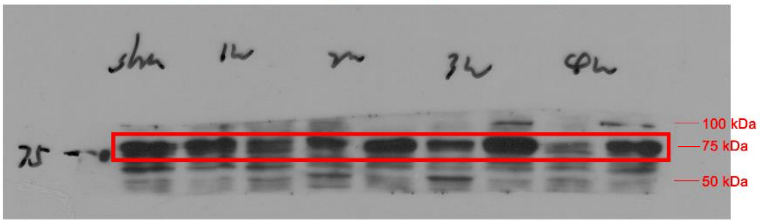

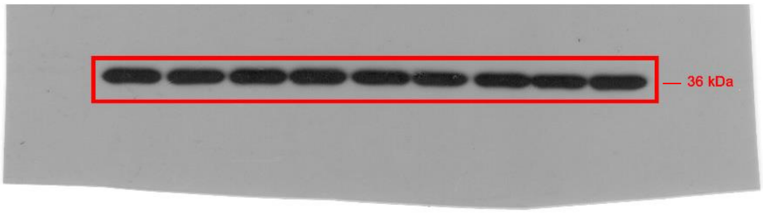

Full unedited blot for Figure 3C GAPDH

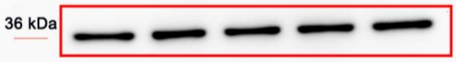

Full unedited blot for Figure 3C p75^{NTR}

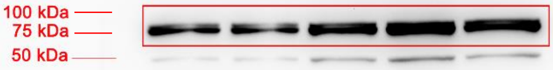

Full unedited blot for Figure 3E GAPDH

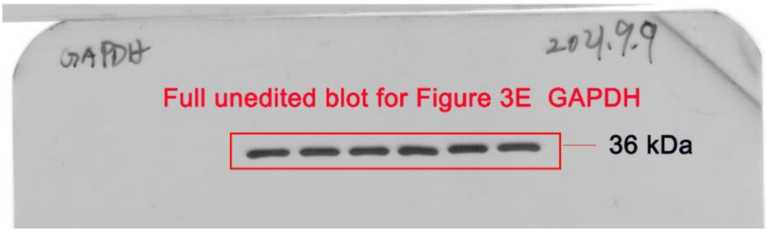

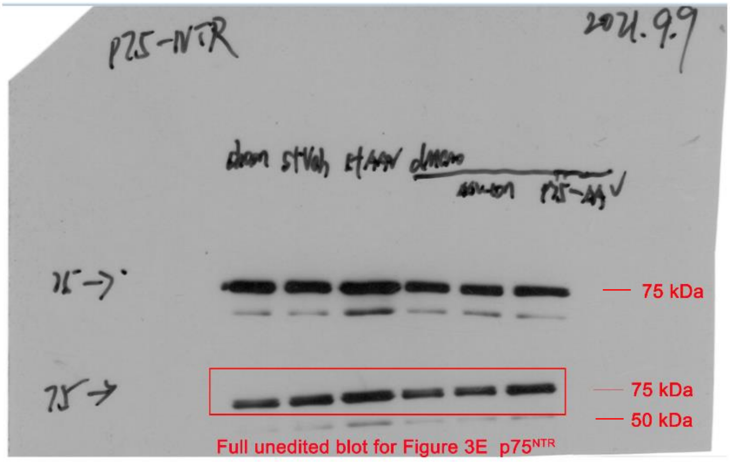

Full unedited blot for Figure 3E p75^{NTR}

Full unedited blot for Figure 4A GAPDH

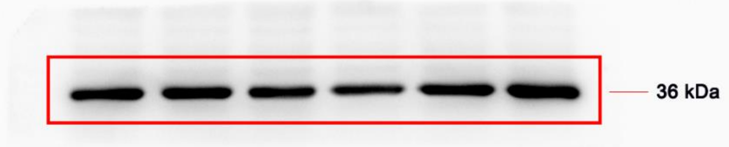

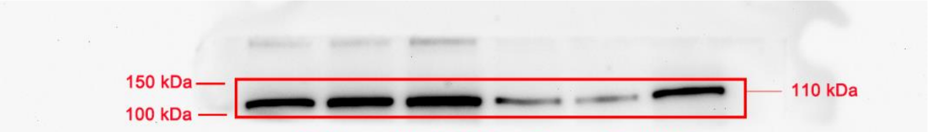

Full unedited blot for Figure 4A HIF-1α

Full unedited blot for Figure 4B HIF-1α

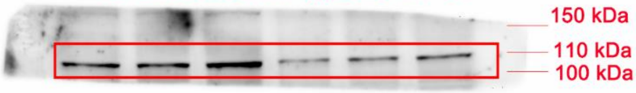

Full unedited blot for Figure 4B Lamin B1

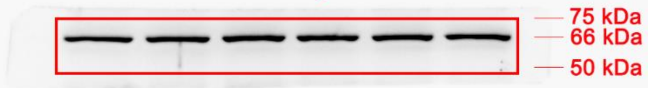

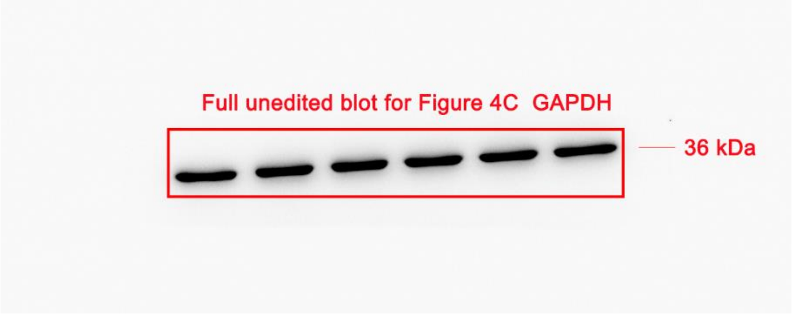

Full unedited blot for Figure 4C GAPDH

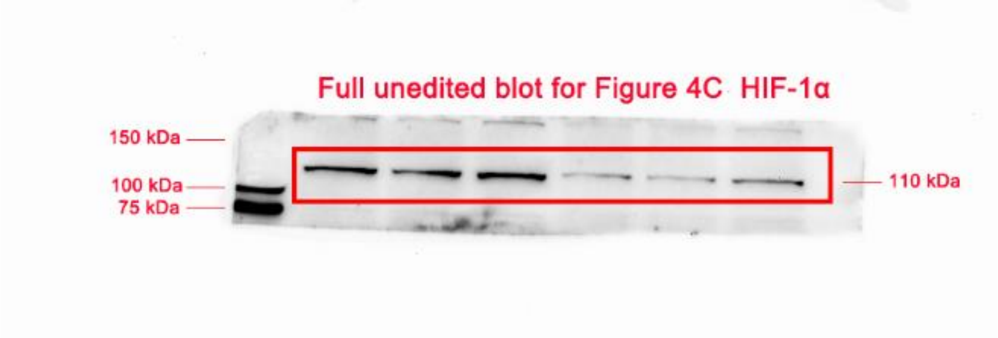

Full unedited blot for Figure 4C HIF-1α

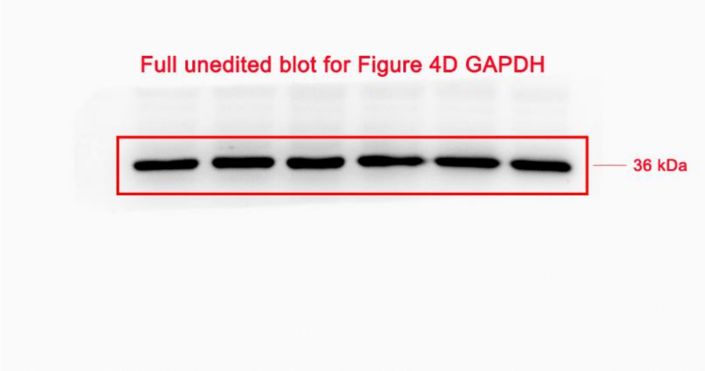

Full unedited blot for Figure 4D GAPDH

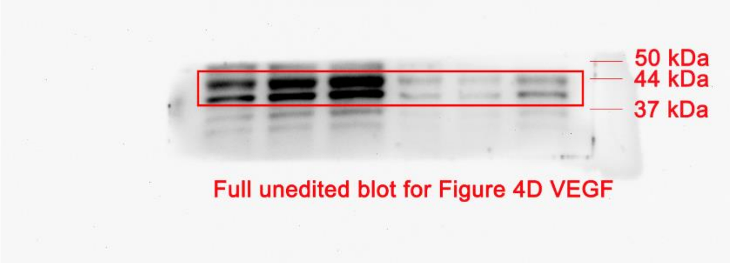

Full unedited blot for Figure 4D VEGF

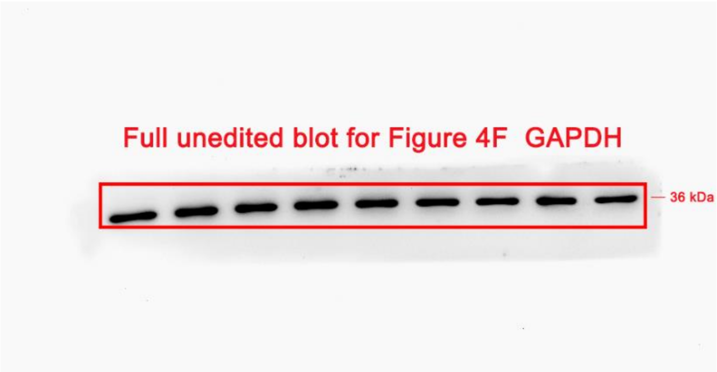

Full unedited blot for Figure 4F GAPDH

Full unedited blot for Figure 4F HIF-1α

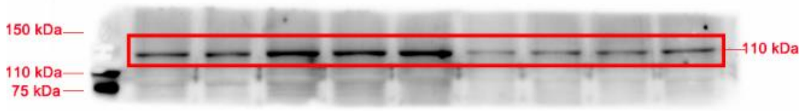

Full unedited blot for Figure 4G GAPDH

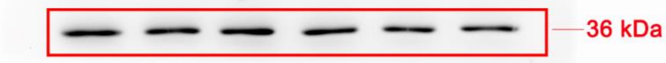

Full unedited blot for Figure 4G VHL

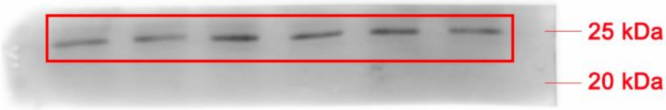

Full unedited blot for Figure 4H p75^{NTR}

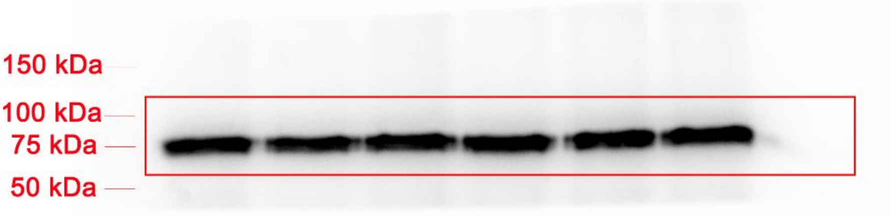

Full unedited blot for Figure 4H VHL

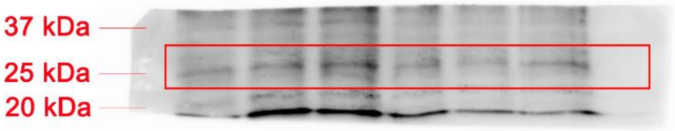

Full unedited blot for Figure 4I HIF-1α

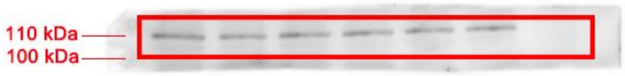

Full unedited blot for Figure 4I K48-Ub

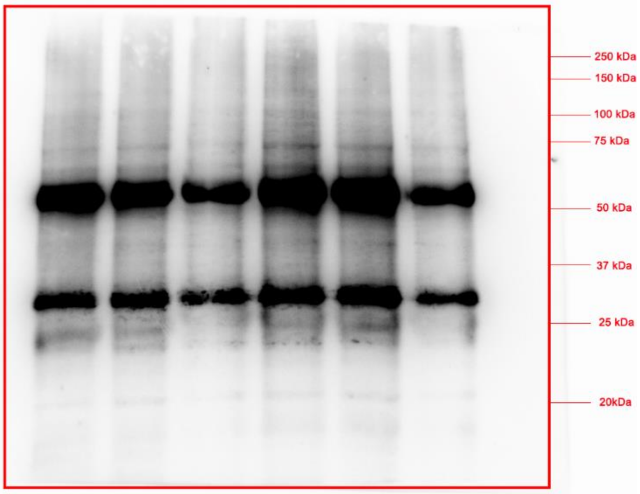

Full unedited blot for Figure 4I VHL

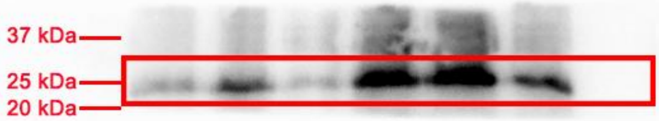

Full unedited blot for Figure 5B GAPDH

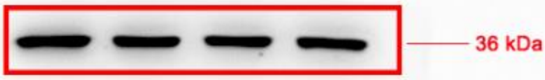

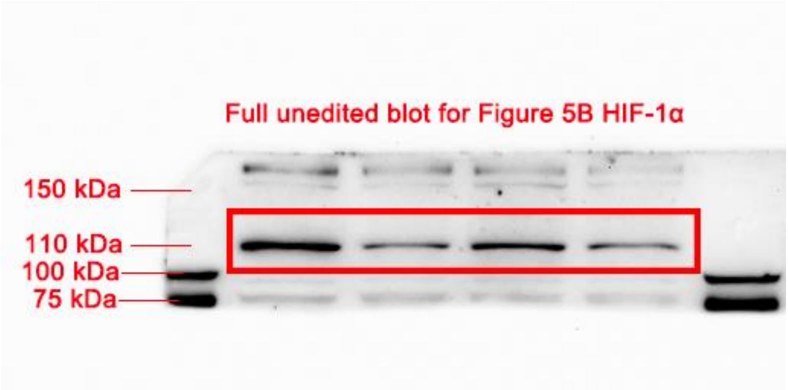
Full unedited blot for Figure 5B HIF-1α
150 kDa
110 kDa
100 kDa
75 kDa
