# Supplementary figures and images for: The p75 neurotrophin receptor attenuates secondary thalamic damage after cortical infarction by promoting angiogenesis
Source: CNS Neurosci Ther. 2024 Jul 28;30(7):e14875. doi: 10.1111/cns.14875 (PMC11284236; doi:10.1111/cns.14875)

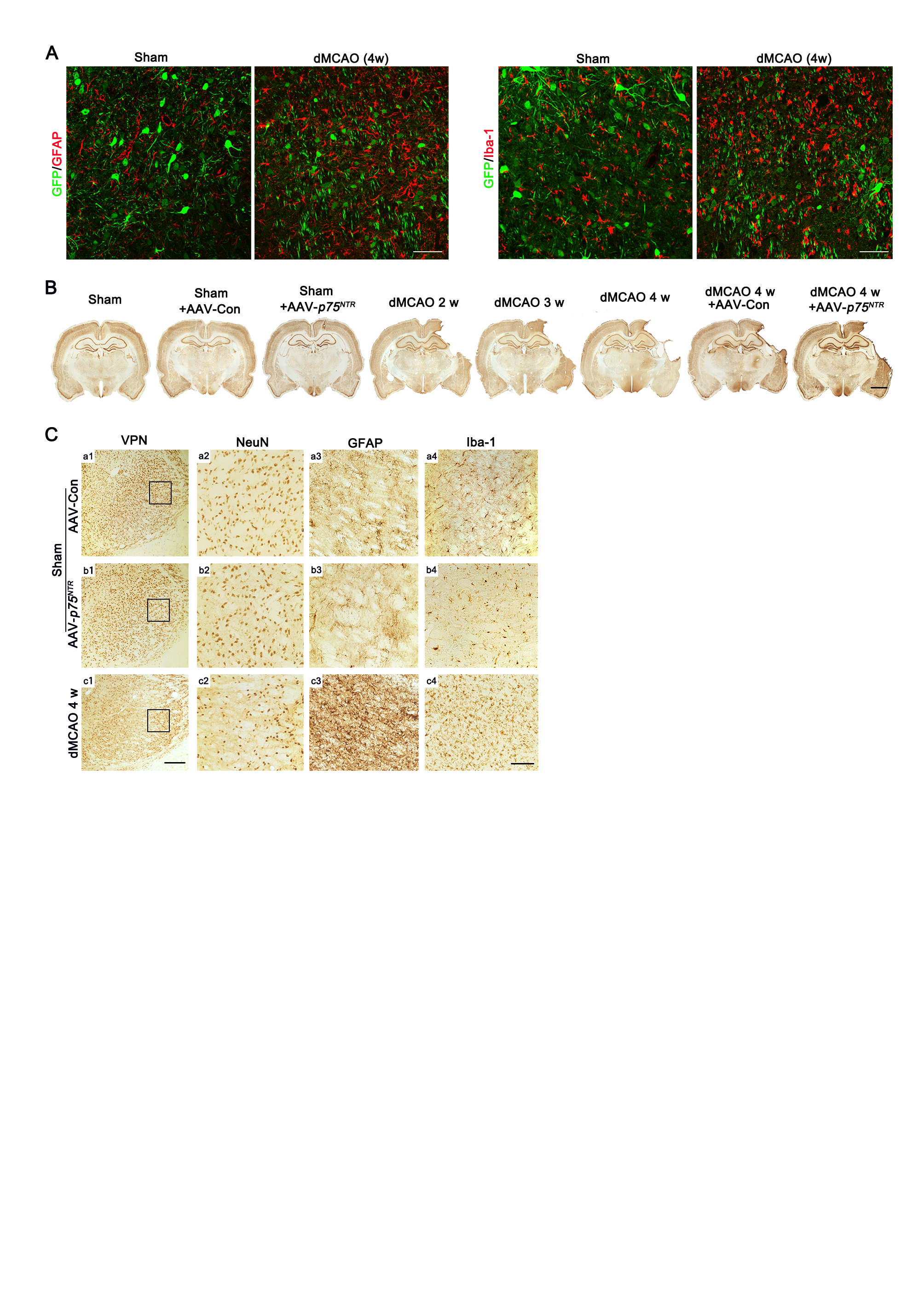

Supplement: Supplementary file 3 — Figure S1. [file CNS-30-e14875-s002.tif]
